# Fine-tuning large language models to generate single-atom catalyst synthesis procedures

**DOI:** 10.1038/s42004-026-02046-y

**Published:** 2026-06-19

**Authors:** Manu Suvarna, Matteo Manica, Fillipo Ficarra, Andres M. Bran, Andrea Ruiz-Ferrando, Magdalena Lederbauer, Philippe Schwaller, Javier Pérez-Ramírez, Teodoro Laino

**Affiliations:** 1 Institute for Chemical and Bioengineering, Department of Chemistry and Applied Biosciences, ETH Zurich, Zurich, Switzerland; 2 NCCR Catalysis, Zurich, Switzerland; 3 IBM Research Europe, Rüschlikon, Switzerland; 4 Laboratory of Artificial Chemical Intelligence, Institute of Chemical Sciences and Engineering, EPFL, Lausanne, Switzerland

**Keywords:** Catalyst synthesis, Theory and computation

## Abstract

Heterogeneous catalyst design is primarily expert-driven and difficult to keep track amidst rapidly growing literature, motivating the use of large language models (LLMs) to learn from existing knowledge. While generalist LLMs show some capability in generating synthesis procedures, they struggle on encountering complex catalytic systems like single-atom catalysts (SAC). Here, we investigate fine-tuning of LLMs to improve the generation of SAC synthesis procedures with chemical relevance. We curate a dataset of 2,964 SAC publications and train the Granite-based LLM to recommend protocols based on user-defined prompts including metal-support combinations, synthesis method, and target reactions. We demonstrate the model’s practicality through a user interface allowing researchers to query procedures tailored to their design conditions. Human-in-the-loop validation of 150 model-generated SAC protocols confirmed coherent, context-aware procedures with synthesis sequence steps that align with experimental logic, along with limitations. We underscore LLM-generated protocols must be critically reviewed and used as assistive tools, and highlight fine-tuned LLMs for AI-assisted synthesis planning driving innovation in catalysis research.

**Figure:**
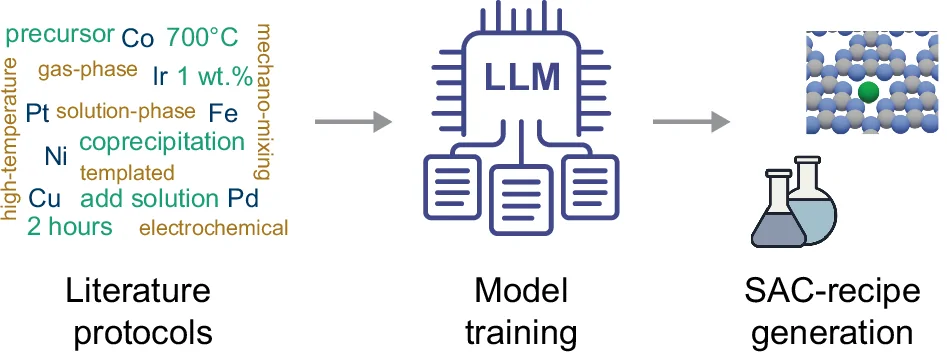

## Abstract (web summary)

Although large-language models can generate synthetic procedures for heterogeneous catalyst design based on the existing body of literature, they struggle when encountering complex catalytic systems such as single-atom catalysts (SACs). Here, the authors curate a dataset of 2,964 SAC publications to train the Granite-based LLM to recommend protocols based on user-defined prompts — including metal-support combinations, synthesis method, and target reactions — and demonstrate the model’s practicality through a user interface that allows researchers to query procedures tailored to their design conditions.

## Introduction

The exponential rise of large language models (LLMs) since 2022 has garnered significant interest in their integration into modern society, transforming how people work, learn, and communicate. Their widespread adoption marks one of the fastest in technology history, reaching millions of users within weeks, surpassing even internet and smartphone-based technologies^1^. LLMs such as GPT, Gemini, Llama, Granite, Mistral, etc. have demonstrated a growing optimism about their potential to transform research workflows in natural sciences and engineering^2–5^. LLMs are capable of learning from vast numbers of research articles to support comprehensive literature reviews, uncover trends in data, and generate synthetic datasets for machine learning (ML) applications^6–10^. In automated laboratories, LLMs can be used to extract knowledge from historical data and generate code and machine-readable instructions to execute tasks, working in collaboration with robotic platforms to enable closed-loop experimentation^11–14^. Additionally, LLM-generated structured and reproducible synthesis outputs support findable, accessible, interoperable, and reusable (FAIR) data principles, improving data standardization^15,16^. Beyond research, these tools also serve as educational aid, helping students and early-career scientists understand existing literature through collective insights across studies and propose potentially underexplored research objectives.

Despite these promising developments, it remains unclear whether the generative capabilities of LLMs can extend to the synthesis of heterogeneous catalysts, which is largely viewed more as an art than a science^17,18^. Heterogeneous catalyst synthesis involves nuanced dependencies among precursor and solvent chemistry, metal-support choice, pre- and post-treatment conditions, sequence of unit operations, and it still relies heavily on expert intuition and design rules^19,20^. These subtleties give rise to a wide variety of protocols and catalyst formulations, reflected in an expansive and continuously growing body of literature^21^. This body of work provides a domain-specific corpus to advance LLMs toward plausible, context-aware synthetic procedures. To address this challenge, we present a systematic attempt to instruct and evaluate LLMs for the generative design of synthesis protocols for single-atom catalysts (SAC). This class of material has garnered significant attention within the catalysis community due to their atomic precision, efficient metal usage, and distinct catalytic behavior^22,23^. Their rapid rise over the past decade reflects a broad interest across thermo-, electro-, and photocatalytic applications^24–26^. However, the diversity of compositions and the proliferation of wet-chemical, high-temperature, gas-phase, and hybrid synthesis strategies, often with incremental variants of known procedures^27–30^, make it increasingly difficult to track developments and validate reported protocols^31^.

**Fig. 1:**
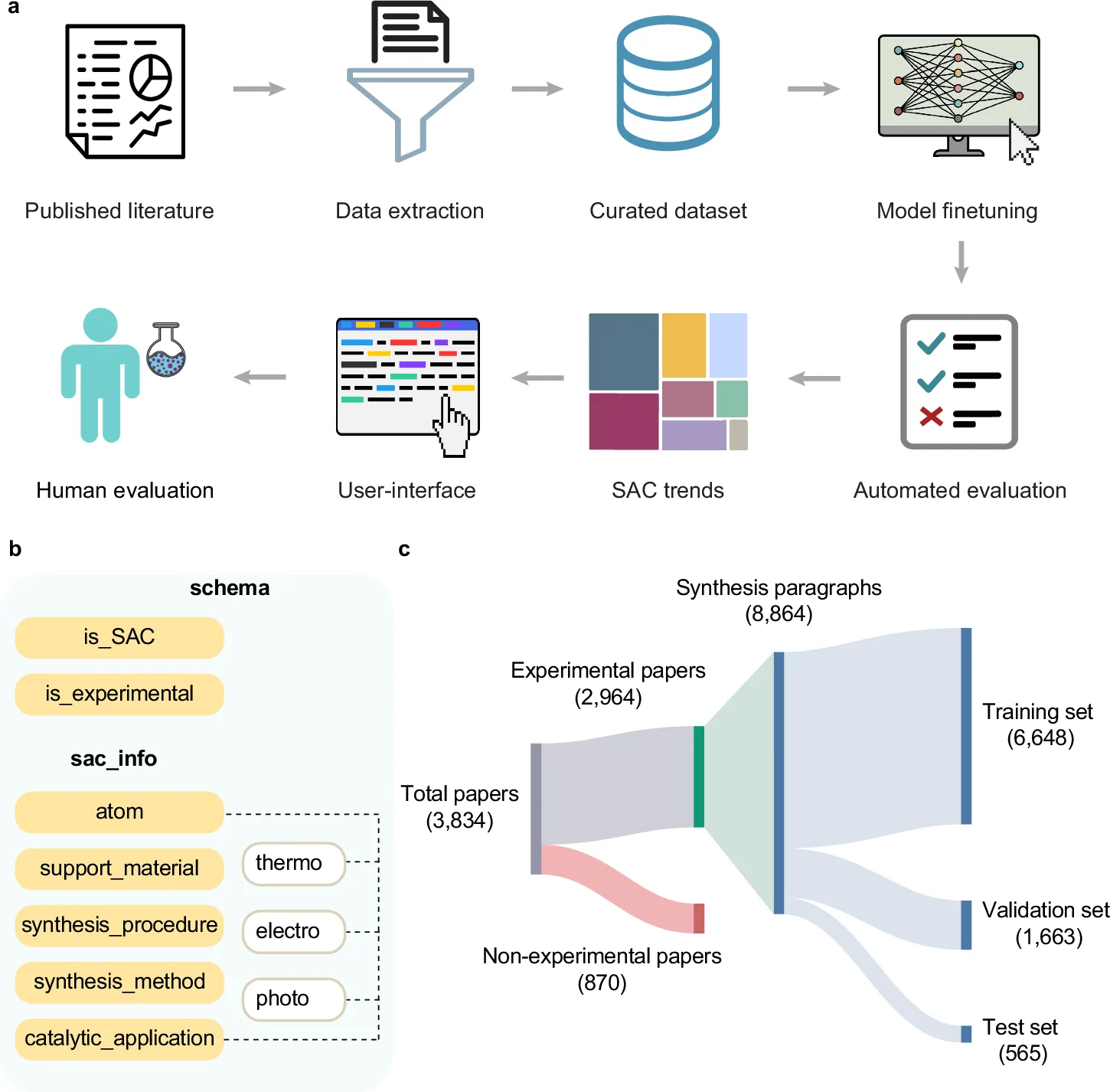
Process schematic of the workflow adopted in the study. **a** SAC literature published between 2010 and 2024 was mined to extract synthesis procedures, which were curated into a dataset for fine-tuning language models. A manually test set and a separate custom dataset were used to validate extracted procedures and to evaluate model-generated outputs using automated metrics. The annotated dataset was further used to identify SAC design trends. The fine-tuned models were integrated into a user interface for interactive generation of candidate synthesis routes, and representative protocols were finally reviewed by domain experts through human evaluation. Icons adapted with permission from ref. ^21^. **b** Each article is first annotated for relevance (is_SAC) and whether it reports experimental work (is_experimental). For relevant entries, detailed synthesis information is captured under the sac_info object, including the metal atom, support material, full synthesis procedure, the synthesis method, and the catalytic application type. **c** Sankey diagram showing the flow of data collection and categorization based on this schema, mapping unstructured text to structured synthesis records for model development.

The current effort builds upon our previously reported study to automate synthesis protocol extraction from published literature^21^. The prior work could not recommend SAC procedures on its own, but rather converted a user-provided full procedure text into structured synthesis actions using a transformer model. Here, we take a clear step forward toward AI-assisted SAC synthesis planning by developing LLMs to generate synthesis procedures de novo from user-defined prompts (e.g., metal-support combination, synthesis method, and target reaction). For this purpose, we develop and evaluate LLMs trained on a curated corpus of 2964 peer-reviewed SAC articles, spanning over 50 synthesis procedures categorized into 8 subgroups, allowing us to critically assess both the opportunities and limitations of LLM-guided catalyst design. Importantly, our emphasis also shifts from data standardization in the previous work to the trustworthiness of the generated recipes in the current study. Herein, we propose multiple automated and human-in-the-loop evaluation metrics to test whether outputs are coherent, context-aware and consistent with experimental logic rather than hallucinated (Fig. 1a). Our analysis reveals that fine-tuning LLMs with SAC-specific knowledge enables generating detailed synthesis protocols based on user-defined prompts, uncovering trends in catalyst design, including metal-support preferences, metal incorporation strategies and preferences in synthetic routes. While the model shows strong generalizability, human-expert review and validation remain essential for reliable application in experimental workflows.

## Results and discussions

### Article collection and annotation

To develop a robust LLM for generating SAC synthesis procedures, we curated a comprehensive and diverse corpus of SAC-related scientific literature published between 2010 and 2024. Relevant articles were retrieved from the Web of Science using targeted keyword queries (e.g., single atom catalyst, low nuclearity catalyst, atomically dispersed catalyst) to ensure topical relevance. The search yielded 3834 peer-reviewed research articles, including both main texts and supplementary information (SI). All the articles, including their SI, were collected in PDF format and compiled together using their unique DOIs. These articles were converted from PDF to raw^32^, machine-readable text using Docling^33^. Docling is a layout-aware text processing tool that retains the structural integrity of scientific content, including section headers, equations, figures, and tables, ensuring high-quality input for downstream information extraction.

A critical step in enabling LLM training for synthesis generation is to precisely and systematically annotate the corpus^34^. Herein, our objective was to extract experimentally relevant information from the publications and annotate them to describe the synthesis of SAC, while filtering out articles that were either computational, review, or focused on other catalyst types (e.g., dual-atom or nanoparticle systems). For this purpose, we developed a three-step annotation methodology leveraging Qwen2.5-72B-Instruct^35^, a high-performance instruction-tuned LLM capable of zero-shot comprehension across long scientific texts (see “Methods”).

The first step involved paragraph-level retrieval using an instruction-tuned LLM. Each article along with its supporting information was converted from PDF to text and processed to identify paragraphs containing experimental details, including: (i) metal atom, (ii) support material, (iii) precursor and solvent information, (iv) synthesis procedure, and (v) target application (thermo-, electro-, or photocatalysis) (Fig. 1b). This approach was necessary because synthetic details are often scattered across the main text and supplementary sections across different publishers, making rule-based or keyword-based extraction ineffective. In the second step, the retrieved paragraphs were parsed into a structured schema with standardized metadata fields. From the original corpus, 2964 articles met the criteria of being experimental and SAC-specific. These were used to generate a manually curated test set of 252 articles (with 565 synthesis procedures). The remaining 2712 articles were split into training and validation sets (80:20), yielding 6648 and 1663 synthesis procedures, respectively (Fig. 1c).

### Large language model and extraction accuracy

#### Fine-tuning the large language model

**Fig. 2:**
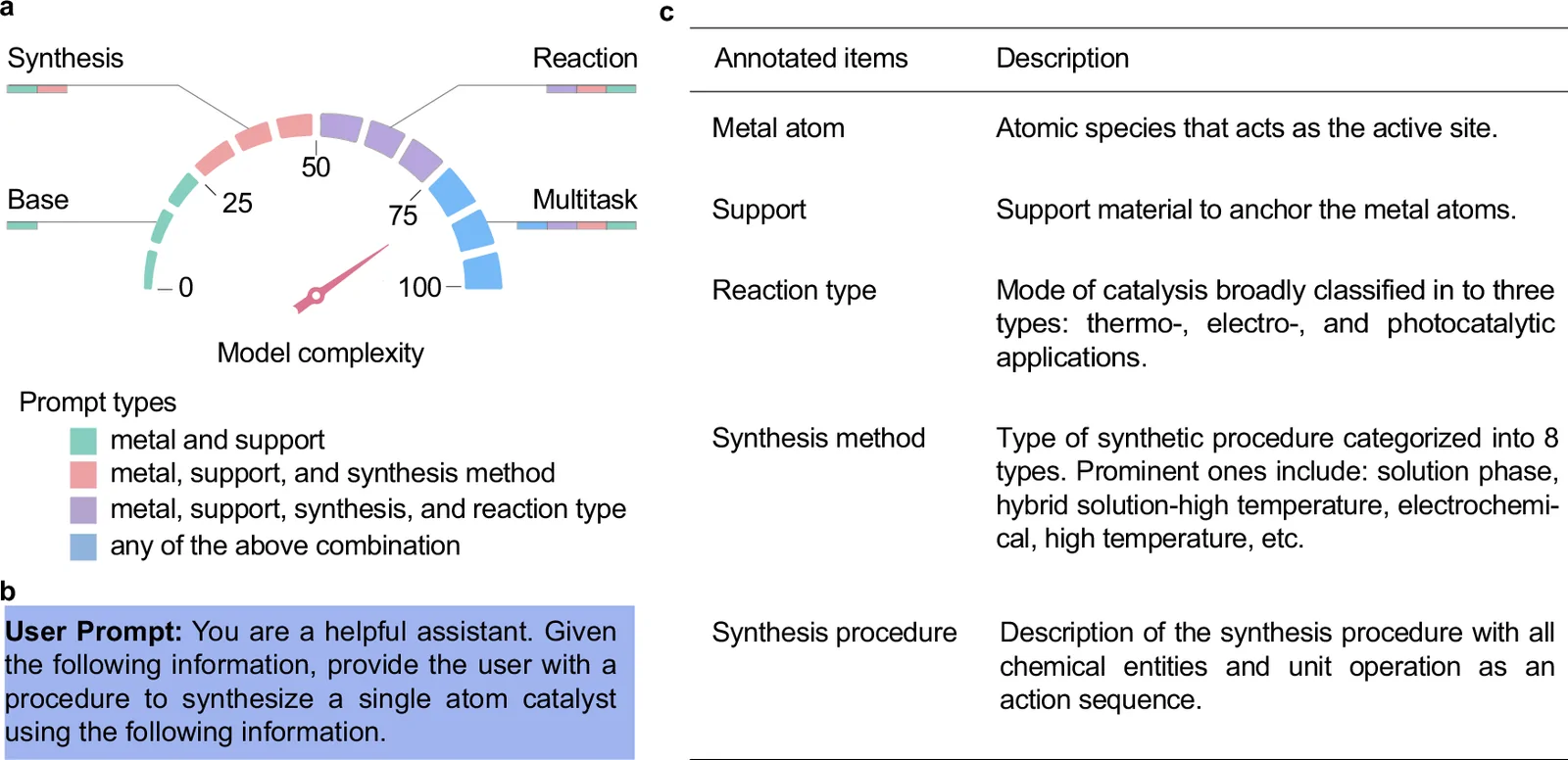
Prompt complexity across different LLM training configurations. **a** Prompt types vary in the amount of structured input provided, from minimal information (i.e., metal and support) to detailed specifications including synthesis method and reaction type. The numbers in the figure are arbitrary and illustrate lower to higher complexity. **b** A consistent system prompt used during model training, adaptable to different prompt configurations. **c** Description of the annotated items that make-up the user-defined prompts.

Following dataset curation, we fine-tuned the IBM Granite 3.2-8B Instruct^36^, a decoder-only transformer model optimized for instruction-following tasks. We selected the IBM Granite 3.2-8B Instruct model due to its strong instruction-following capabilities, open-weight availability, and suitability for domain-specific fine-tuning, enabling robust control over training, evaluation, and reproducibility, features not readily accessible with other commercial models. Fine-tuning LLMs for domain-specific use requires more than retraining on specialized data, and also involves adding new knowledge while preserving the model’s existing capabilities^37^. This is particularly challenging in the domain of heterogeneous catalysis, where training models from scratch could be arduous due to limited access to large and well-curated datasets and high and subsequent computational costs. Herein, two fine-tuning strategies were employed: (i) full fine-tuning, wherein all model parameters were updated for maximal domain adaptation, and (ii) LoRA^38^, a memory-efficient method that updates a small set of parameters within attention layers (see “Methods”). We developed four distinct model variants based on the specificity of input prompts (Fig. 2a). The base model requires minimal input, typically the metal and support information, and is designed for generic synthesis prediction. The synthesis model adds a preferred synthesis route to the base inputs, enabling targeted synthesis. The reaction model includes additional context such as the driving force (e.g., thermo-, electro-, or photocatalysis), reflecting reaction-driven synthesis planning. This model progression, i.e., from simple to highly specific inputs, mirrors real-world experimental needs, where the user may request a generic SAC synthesis, e.g., synthesis of platinum single-atoms on carbon support (Pt/C); or specify precise and detailed input, such as synthesis of iron single-atoms on nitrogen-doped carbon support (Fe/NC) for CO_2_ electroreduction via solution-phase synthesis method. Lastly, a multitask model is trained across all input types to generalize across prompts defined by the user. For example, if the user specifies a synthesis type for a specific metal without listing the support, or a metal-support combination for a specific reaction, without prompting, the synthesis method would be categorized under multitasking. All models were trained using a consistent system prompt to offer model generality (Fig. 2b). Details of the catalysis-related terms used to instruct the model are depicted in (Fig. 2c).

#### Extraction accuracies of the synthesis procedures

After fine-tuning, we assessed how accurately Qwen2.5-72B-Instruct was able to extract SAC synthesis information from published articles, when compared to the test set. This test set consisted of 252 articles that were manually reviewed and annotated by domain experts, resulting in 565 unique synthesis procedures. The manual annotation ensured that relevant information in the synthesis description, including atomic species, support material, catalytic application, and procedural details, was correct to serve as the ground truth. When applied to the same set of articles in the test set, Qwen2.5-72B-Instruct extracted a total of 649 synthesis procedures. The higher number of procedures compared to the manually curated counterpart could arise because several articles describe multiple experimental variations, for example, investigating multiple metal atoms while keeping the rest of the procedure similar, or screening multiple supports while studying a given metal atom^21^. The textual similarity between the LLM-generated and human-annotated synthesis procedures was evaluated using metrics including the BiLingual Evaluation Understudy (BLEU) score, Levenshtein similarity, and mean length difference (MLD) scores^21,34^ (see “Methods”). The model achieved scores of 0.62, 0.51, and 0.023 on the Levenshtein, BLEU, and MLD metrics, respectively (Supplementary Note 1). Taken together, these three metrics offer a first, triangulated assessment of how closely the LLM output approximates real-world synthesis descriptions.

**Fig. 3:**
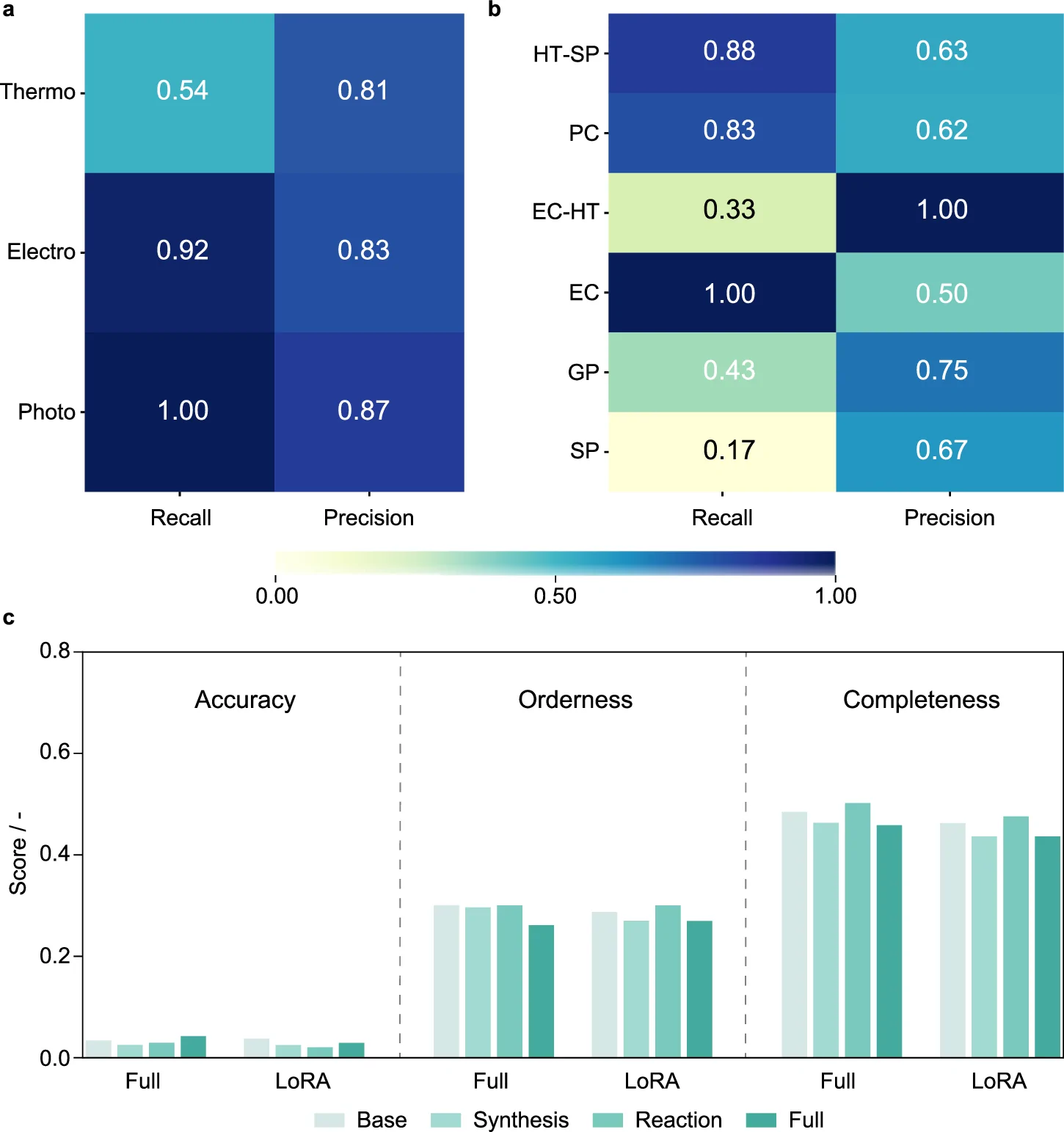
Predictive capabilities of the LLM. Heatmaps depicting extraction accuracy in terms of recall and precision for **a** catalysis type and **b** synthesis methods, based on comparison with manually annotated test data. Abbreviation used include SP solution-phase, GP gas-phase, EC electrochemical, PC photochemical and their corresponding hybrid methods. Evaluation of the full fine-tuned and LoRA-adapted models for synthesis generation on the benchmark dataset. Performances are reported across three task-specific metrics, i.e., accuracy, order retention, and completeness, and both models achieve comparable scores. **c** Evaluation of the full fine-tuned and LoRA-adapted models for synthesis generation on the benchmark dataset. Performances are reported across three task-specific metrics, i.e., accuracy, order retention, and completeness, and both models achieve comparable scores.

In addition to evaluating full-text similarity, we assessed the LLM’s ability to correctly identify structured categorical information, specifically the catalysis type and synthesis method associated with each SAC procedure^39^. For this purpose, we used the recall (percentage of true cases the model correctly identified) and precision (percentage of predictions that were correct) metrics (Fig. 3a, Supplementary Note 2, and Supplementary Table 1). The model achieved high recall and precision (>0.8) for electro- and photocatalysis, likely due to consistent and explicit phrasing in the literature, such as “used for hydrogen evolution reaction (HER) in alkaline media” or “visible-light driven CO_2_ reduction.” In contrast, thermocatalysis showed lower recall (0.54), mainly because these reactions are often not explicitly labeled in the literature and must be inferred from experimental context. For instance, reactions like CO oxidation or CO_2_ hydrogenation may be thermocatalytic but not labeled as such, making them difficult for the model to classify without clear cues. A domain expert may infer this from operating conditions (e.g., 300 °C, 50 bar H_2_), but a language model trained on explicit textual patterns may misclassify such cases, especially when similar reactions are also studied under electrochemical conditions.

On the synthesis method classification (Fig. 3b and Supplementary Table 2), the model performed well on recall for hybrid solution-phase and high-temperature (0.88) and photochemical (0.83) methods, where the former is frequently reported in literature and the latter comprises identifiable terminologies such as “photochemical,” “light mediated,” etc. in the synthesis paragraphs for the model to learn from. Electrochemical and gas-phase synthesis also performed reasonably well, likely because of their distinct keywords (e.g., “electrodeposition,” “CVD,” “plasma”) that recur across papers and aid in reliable recognition. Notably, the model failed to identify high-temperature synthesis, resulting in a recall and precision of zero. This is likely due to two reasons. First, a minor fraction of the test set (<5%) comprised high-temperature synthesis. Second, even when high-temperature methods were present, they were often included within hybrid procedures that involved solution or precursor treatments. As such, distinguishing between a purely high-temperature process and a hybrid requires subtle contextual understanding, for example, recognizing that high-temperature heating occurs without any prior solvent-based step. This type of reasoning can be intuitive for human chemists but is challenging for language models unless the distinction is made explicitly in the text^13,39^.

### Evaluation of the synthesis procedure generation

Following the annotation evaluation, we assessed the LLM’s ability to generate complete SAC synthesis procedures based on user prompts. This was performed using both the full fine-tuned and LoRA-based parameter-efficient tuning methods, as described in the “Methods” section. For consistency, we compared the model-generated SAC synthesis procedures to the annotated test set using evaluation metrics including Levenshtein, BLEU, and MLD scores^21,34^. To assess how prompt specificity influences the quality of SAC synthesis generation, we evaluated the full and LoRA fine-tuned models, along with comparison with non-fine-tuned counterparts, under all four distinct prompts (Table 1). Here, we note that metrics quantify whether different fine-tuning strategies lead to more coherent, procedural, and structurally consistent text, rather than to judge whether the generated procedures are chemically meaningful, a task that is left to the other evaluation protocol (described in Section “Human-informed chemical validation strategies”). Given the scale of the test set (565 paragraphs), manual assessment was not feasible at this early stage, and these automated quality checks served as early indicators before applying more meaningful downstream evaluations.

**Table 1:** Prediction metrics on the full fine-tuned model and on the LoRA fine-tuned models, and comparison with non-fine-tuned counterparts

Fine-tuning	Model type	^a^Mean Levenshtein score	^a^Mean BLEU score	Mean length difference
Full	Base	0.40 (0.31)	0.32 (0.21)	0.32
Full	Synthesis	0.41 (0.32)	0.32 (0.23)	0.14
Full	Reaction	0.40 (0.29)	0.31 (0.19)	0.27
Full	Multitask	0.41	0.32	0.14
LoRA	Base	0.41 (0.31)	0.32 (0.21)	0.14
LoRA	Synthesis	0.39 (0.32)	0.30 (0.23)	0.42
LoRA	Reaction	0.40 (0.29)	0.31 (0.19)	0.26
LoRA	Multitask	0.39	0.28	0.34

^a^Values in parentheses represent the corresponding model performance before fine-tuning.

The full-fine-tuned model achieved consistent performance across all prompt types, with Levenshtein scores of 0.40–0.41 and BLEU scores of 0.31–0.32. The lowest MLD was observed for the synthesis prompt (0.14), suggesting that including the synthesis method in the prompt allows the model to generate procedures of appropriate length, closely matching the reference. While adding the reaction type slightly increased the MLD (0.27), it also improved Levenshtein similarity (0.41), indicating better alignment in actual content. This implies that more detailed prompts, especially those tied to the intended catalytic application, help the model produce text that resembles human-written procedures, even if the length varies slightly. For the LoRA-tuned models, Levenshtein and BLEU scores were comparable to the full models, but MLD values were notably higher, especially under minimal prompts. This indicates the LoRA models tended to over- or under-generate synthesis text when given limited input. More detailed prompts (e.g., with synthesis method and catalysis type) helped reduce this variability, but overall, LoRA tuning showed less control over procedural length, making it less reliable for precise synthesis generation without stronger prompt guidance.

### Customized evaluation on the benchmark dataset

In our initial evaluation on 565 annotated synthesis paragraphs, we assessed the full Granite model and its LoRA variant using text-overlap measures such as BLEU, Levenshtein, and MLD. While these scores quantify surface-level similarity between the generated and ground truth procedures, they may remain inadequate to capture the chemistry-related elements of a synthesis, including but not limited to the correct choice of metal precursors, support materials, or the logic and ordering of experimental steps. To enable more domain-relevant validation, we implemented a custom evaluation suite that consists of a manually curated dataset (see “Methods”). The model input queries were generated using a standardized template described in Section “Fine-tuning the large language model” and Fig. 2b. Given such a query, the procedures were generated by the fully fine-tuned Granite model and the LoRA fine-tuned model which we compare directly with the corresponding ground-truth synthesis using three complementary metrics: accuracy (strict match of step details), order (correct sequencing of matched steps), and completeness (fraction of ground-truth steps recovered).

The results for the benchmark dataset are summarized in Fig. 3c. Notably, full procedure accuracy was low (<10%) for both models, reflecting the challenge of generating steps that exactly reproduce the literature protocols at a key-wise level. Furthermore, as SAC and heterogeneous catalysts in general could be synthesized via multiple valid recipes rather than a single correct protocol, the LLMs could generate alternate versions of the ground truth, thus leading to lower scores. Order retention scores were higher, typically around 25–30%, indicating that while exact procedure reproduction is challenging, the models generally follow the logical sequencing of synthesis unit operations. This suggests that outputs are not arbitrary lists of actions but maintain a plausible procedural structure. Completeness emerged as the strongest metric, with models recovering 45–55% of ground-truth steps. Overall, the results show that fine-tuned and LoRA models perform comparably.

Several factors likely contribute to the overall gap between generated and ground-truth procedures. First, variability in literature reporting means that not all steps are consistently described, and strict key-wise evaluation penalizes minor differences in terminology or language granularity. We have discussed these aspects in length in our earlier work^21^. Second, parser limitations may introduce errors during the extraction of structured actions, leading to mismatches when compared to the model output itself. For instance, a ground-truth step such as “Chloroplatinic acid was dissolved in DI water” should be equivalent to a model output of “Add H_2_PtCl_6_ to water,” but if the parser fails to map both names to the same entity; this is counted as an error. Similarly, differences in step granularity can create mismatches: if the ground truth reports “Dissolve the precursor in ethanol. Stir for 12 h” as two separate steps, while the model outputs “Add precursor to ethanol and stir for 12 h” is parsed as a single combined step, the evaluation registers a missing step despite the chemical procedure being correct. Such parser-induced discrepancies and variability in chemical terminologies, and least to mention variations in synthesis procedures, illustrate that low accuracy scores may partly reflect extraction artifacts, and not solely the model’s inability to reproduce valid synthetic procedures. Lastly, we emphasize that the evaluation on the custom benchmark is intentionally stringent, compared to the test-set assessment in Section “Evaluation of synthesis procedure generation”. We adopted this design because synthesis protocols are highly sensitive to small deviations; for example, missing washing or drying steps, swapping impregnation and calcination, or omitting critical post-treatment conditions can materially alter the resulting catalyst structure. Accordingly, low-to-moderate scores under this configuration can be expected and should be interpreted as a conservative lower bound on exact procedural reproduction. They should not be taken as a definitive measure of synthesis quality, especially because multiple valid synthesis routes may exist for a given metal-support combination-which is an inherently open-world problem.

### Insights on SAC research trends

**Fig. 4:**
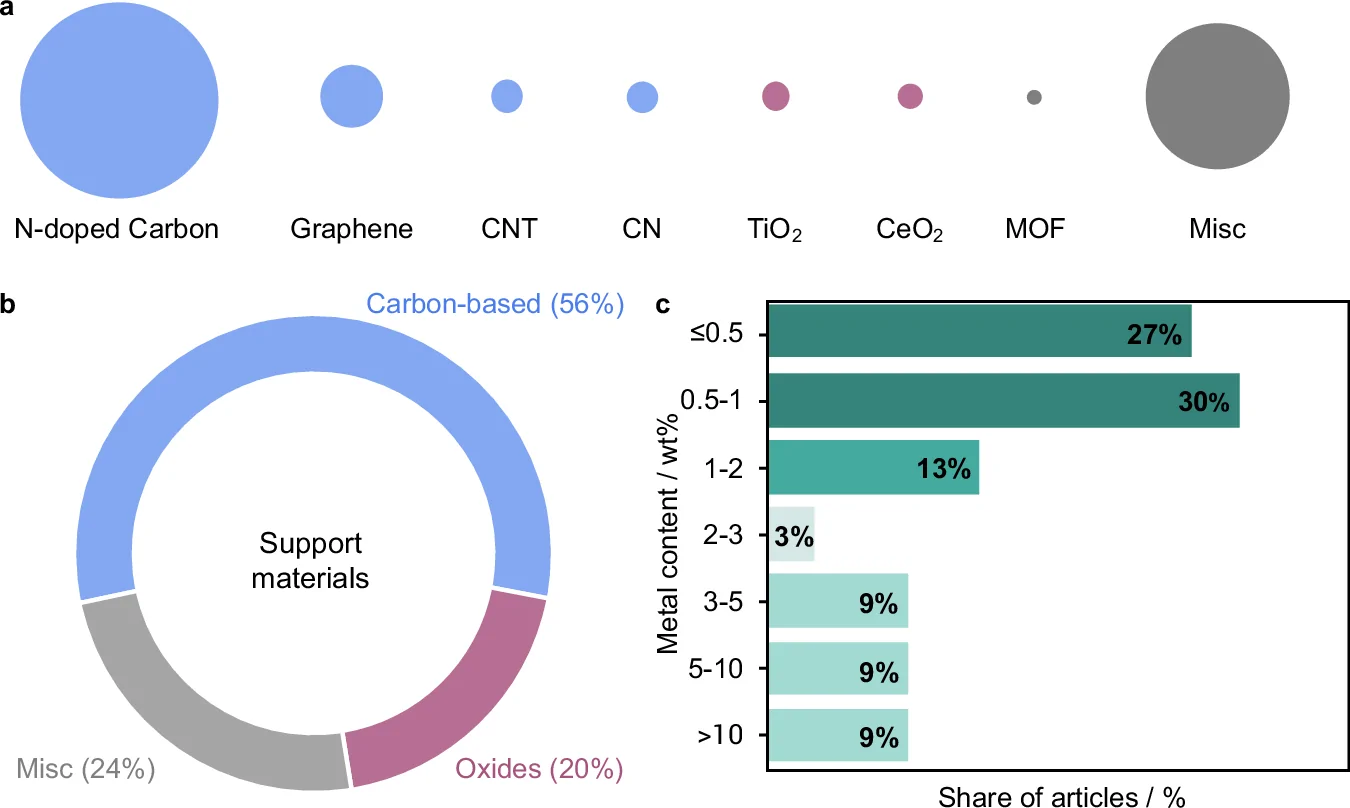
Statistical trends in SAC synthesis protocols. **a,** **b** Summary of support materials and metal content trends from manually annotated articles. The circle diameter in (**a**) corresponds to the normalized count of the support materials. CNT carbon nitrides, CN carbon nanotube, MOF metal organic framework, Misc miscellaneous. The graphene supports depicted include nitrogen-doped and their oxide variants as well. Carbon-based supports dominate (56%), with oxides (20%) and miscellaneous types (24%). **c** Most reported SACs feature metal content below 2 wt%, consistent with the motivation of achieving atomic dispersion.

A detailed statistical analysis of the manually annotated SAC synthesis procedures from the test set revealed distinct trends in the choice of support materials and metal contents. Carbon-based supports dominate, featuring in 56% of the procedures, with nitrogen-doped carbon as the leading choice (Fig. 4a, b). Other widely used carbon variants include graphene, carbon nanotubes, and carbon nitrides. Oxide supports made up 20%, with cerium oxide (CeO_2_) and titanium dioxide (TiO_2_) being the most frequently reported. The remaining 24% fall into a miscellaneous category, encompassing metal organic frameworks (MOFs), organic-inorganic hybrid materials, and carbon materials with inconsistent or rarely reported nomenclature, making them difficult to classify. These findings underscore the reliance on carbon supports given their textural, electronic, and metal-coordination properties^26^.

A strong preference for low metal content was observed, aligning with the goal of achieving atomic dispersion (Fig. 4c). Nearly 70% of procedures report below 2 wt% metal content, with ≤0.5–1 wt% being the most common. Notably, 18% of procedures feature ≥5 wt%, pointing to the evolving trend toward pushing atomic dispersion limits by tailoring synthetic approaches^40^. An analysis of synthesis strategies also revealed preferences linked to specific catalytic applications. For example, solution-phase and hybrid solution-phase and high-temperature methods dominate in electrocatalysis, reflecting their compatibility with metal/NC architectures. In contrast, thermocatalysis exhibits a broader distribution of synthesis routes, including high-temperature, gas-phase, and multi-step hybrid methods, suggesting more diverse design approaches. The photocatalysis subdiscipline shows focused but limited associations, most notably with photochemical and solution-phase synthesis, indicating a smaller yet emerging area of SAC research. Overall, these insights highlight prevailing design practices and can guide both the optimization of well-established metal-support combinations and synthesis routes, as well as the systematic exploration of underexplored pairs and preparation strategies.

### Synthesis generation for user application

To demonstrate the practical utility of our LLM and to allow expert human validation, we developed a user-friendly interface that allows researchers to input structured prompts and receive complete SAC synthesis procedures. The tool is designed keeping experimental catalysis researchers in mind, particularly those looking to explore untested material combinations, adapt literature protocols, or accelerate hypothesis testing without extensive manual testing and experimentation.

**Fig. 5:**
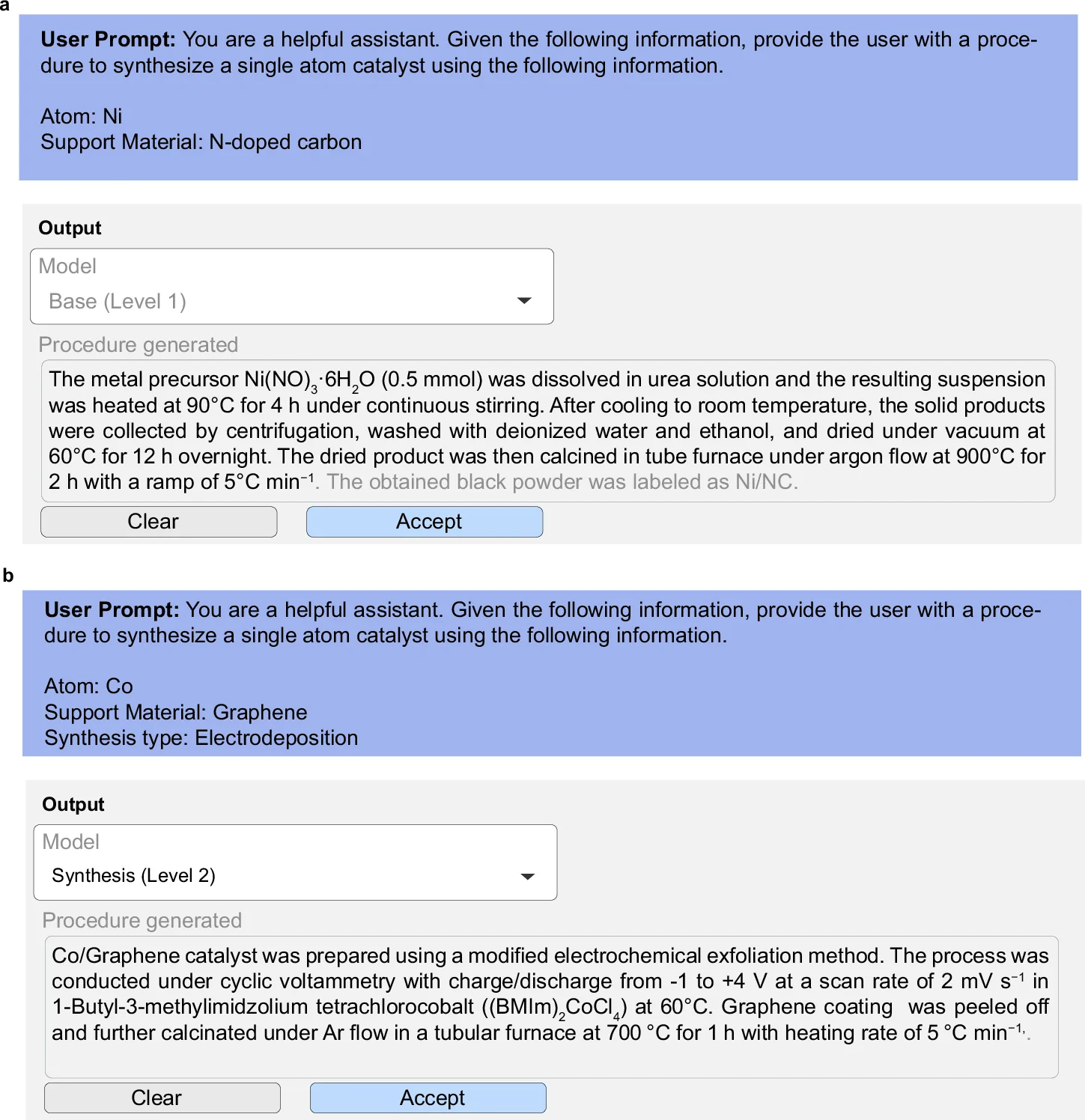
User interface of the LLM output. Representative examples of synthesis procedures generated by the LLM in response to prompts with varying levels of specificity. **a** A basic prompt including only the atom and support material. **b** Detailed prompt that additionally specifies the synthesis method, resulting in a more tailored procedure.

As shown in Fig. 5, users can specify inputs such as the metal atom, support material, and optionally the synthesis method or catalysis type, depending on the level of detail required. The model responds with a coherent synthesis procedure tailored to the input constraints. For example, as shown in Fig. 5a, providing only “Ni” and “N-doped carbon” as prompts to the model leads to the generation of a urea-assisted hybrid solution-phase and high-temperature protocol consistent with widely adopted procedures to design M/NC architectures as discussed previously. As shown in Fig. 5b, prompting with “Co,” “Graphene,” and “Electrodeposition” yields a more targeted electrochemical exfoliation strategy involving ionic liquids and post-treatment, showcasing the model’s ability to recommend nuanced, method-specific protocols. It is worth highlighting that, rather than retrieving fixed literature text, the model generates synthesis procedures based on the user prompts and catalyst design criteria. This capability makes it especially useful in settings where researchers may want to investigate synthesis strategies for underexplored metal-support combinations, tailor procedures to available lab resources (e.g., avoiding high-temperature steps), or inspire unique synthetic approaches through hybrid or unconventional routes.

It is worth highlighting that, while these generated procedures are syntactically coherent and seem chemically plausible even for a domain expert, their authenticity and accuracy require validation. Unlike human-authored protocols grounded in experimental validation, LLM-generated recipes may generate false synthesis steps, present incompatible conditions, or misrepresent chemical feasibility, for instance, in the formation of atomically dispersed Pt species, suggesting the dissolution of H_2_PtCl_6_ using ethanol as the solvent, which can lead to premature reduction and aggregation, undermining the formation of Pt atoms. Such risks have been referenced in recent literature^7,41^, where LLMs trained on scientific text have produced convincing but chemically invalid outputs, especially when prompted with sparse or novel input combinations. This underscores the need for expert review and validation by cross-checking with known literature or experimentally testing the proposed synthesis.

### Human-informed chemical validation strategies

As the application of LLMs in scientific research remains in its early stages, a fully autonomous workflow for generating a catalyst synthesis procedure must be treated with caution^11,42^. Aligning with this motivation and based on our discussions following Sections “Customized evaluation on benchmark dataset” and “Synthesis generation for user application,” we envision a human-in-the-loop validation that enables domain experts to systematically evaluate, refine, and guide model-generated synthesis protocols. These efforts include domain-aware filters, including but not limited to verifying whether precursors and solvents used are compatible, if the unit operation steps follow logical sequences, and thermal treatment conditions align with established principles of SAC synthesis.

**Fig. 6:**
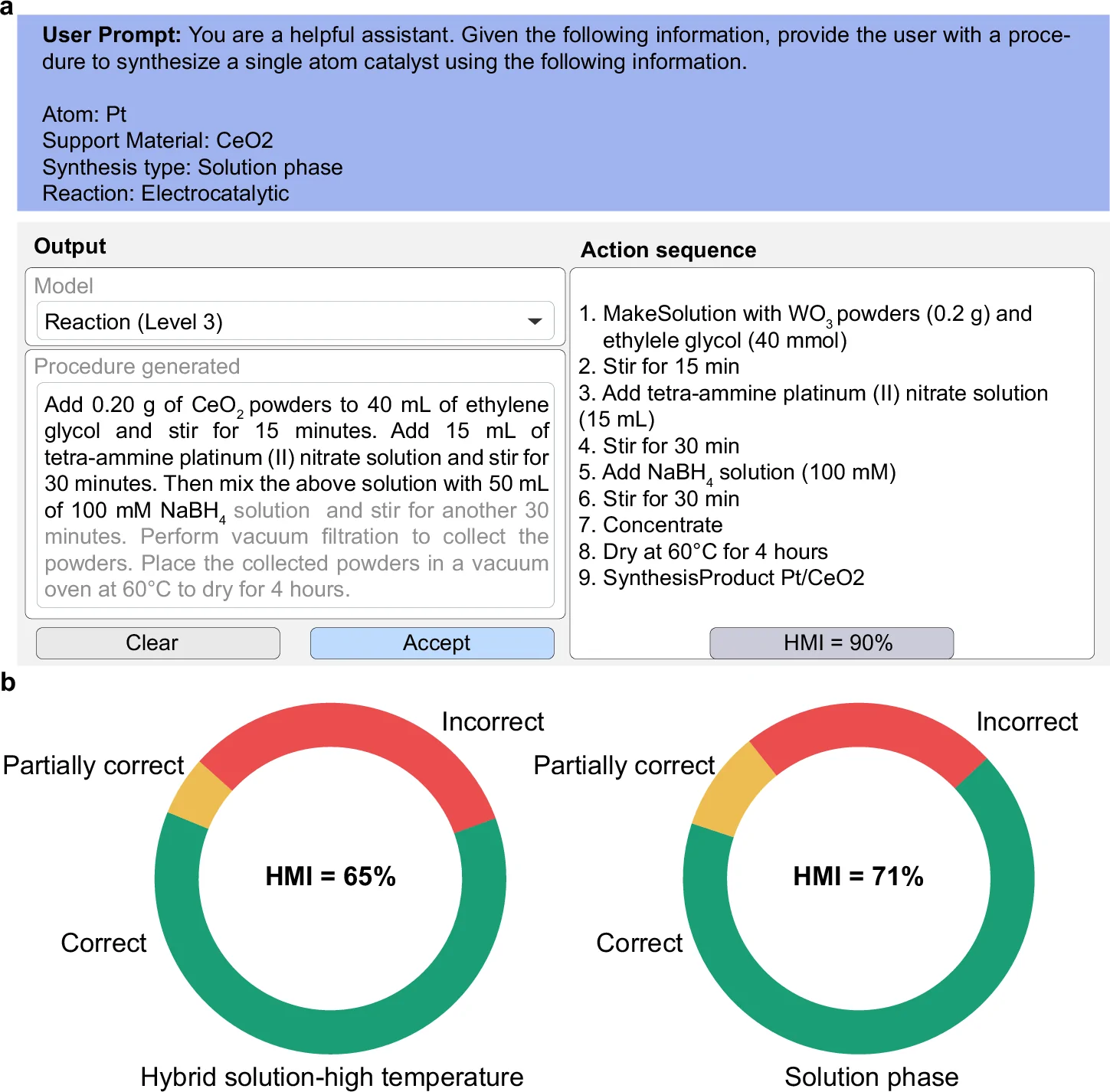
Overview of the human validation on LLM output. **a** Detailed example demonstrating the specific prompts results in a more tailored procedure, followed by the calculation of *HMI* score by domain experts. **b** Model performance using the *HMI* score on the 50 respective protocols generated for hybrid solution-high temperature and solution phase synthesis methods. The color codes used include red, yellow, and green for incorrect, partially correct, and correct predictions, respectively.

For this purpose, we employed the human machine-readability index (*HMI*) metric inspired by our previous work^21^ to assess the robustness of fully fine-tuned models in generating SAC synthesis protocols. It is worth acknowledging that the *HMI* score is calculated by the domain expert after manually reviewing the generated protocols, assessing their compliance with established heuristics of SAC synthesis and chemistry awareness (see “Methods”). We devised prompts encompassing ten metals (Pd, Pt, Fe, Co, Ni, Cu, Mn, Ir, Rh, and Ru) anchored on five different supports (nitrogen-doped carbon, carbon nitride, graphene, CeO_2_, and TiO_2_) and synthesized via three routes reported in the literature, i.e., hybrid solution-high temperature, solution phase, and electrochemical methods. The selection of these metal-support combinations and synthesis routes was informed by their prevalence in the literature, discussed in Section “Insights on SAC research trends.” With ten metals on five different supports and three synthesis routes, this resulted in 150 (10 × 5 × 3) unique prompts. Based on these prompts, the synthesis variant of the fully fine-tuned model was used for generating the procedures. Each recipe contained 4–6 discrete steps on average, as shown in a representative example in Fig. 6a. To aid the readers, we include case studies illustrating common scenarios and demonstrate how domain experts applied *HMI* and interpreted the resulting scores in Supplementary Note 3.

For the hybrid solution-high temperature and solution phase routes, this corresponded to 238 and 228 individual synthesis steps, respectively. In the hybrid solution-high temperature route, 147 steps were observed to be fully correct with respect to process parameters, chemical compatibility, and logical sequencing of unit operations. The remaining steps comprised 13 partially correct and 78 incorrect instances (including hallucinations), yielding an overall *HMI* score of 65%. Similarly, in the solution phase route, 153 of the 228 total steps were fully correct, with 21 partially correct and the remaining 54 incorrect, corresponding to an *HMI* score of 71%. A comparative analysis of the *HMI* with respect to both the synthetic routes is depicted in Fig. 6b. Manual evaluation of the electrochemical synthesis procedures generated by the full fine- tuned model resulted in suboptimal performance. Critical experimental details such as electrode type, calibration protocols, and cycling parameters were often incomplete or incorrectly specified. This limitation can be primarily attributed to the scarcity of electrochemical synthesis data in the model’s training set, since such procedures constitute less than 10% of the annotated literature corpus. Consequently, model predictions and extractions for this specific synthesis method should be interpreted with caution. In contrast, the hybrid solution-high temperature and solution phase methods are among the most frequently reported synthesis routes in SAC literature. Their prevalence in the training data enabled the model to capture procedural nuances and learn recurring patterns, resulting in comparatively higher *HMI* scores, usually in the range of 70%.

Based on the human-in-the-loop validation, we observe that the extracted synthesis protocols frequently capture a logical and coherent sequence of steps, starting from selecting suitable precursors to solution preparation, thermal treatment, and post-processing, thereby reflecting an underlying awareness of common SAC preparation practices. Notably, analysis of incorrect predictions reveals interesting and recurring patterns. Most errors involve the omission of one or two action steps, or the suggestion of process parameters that could be rectified through domain expertise. However, a frequent issue observed is the absence or misreporting of temperatures (*T*) across various synthesis steps. From a SAC chemistry perspective, such inaccuracies require careful attention, as, for example, low *T* (<473 K) can hinder proper support formation, while overly high calcination *T* (>923 K) can promote metal agglomeration or catalyst decomposition, ultimately preventing SAC formation^43–45^. Other common errors include omitting entire procedures such as support synthesis, introducing the metal precursor before support preparation and thereby disrupting the material synthesis sequence, or recommending incompatible or misassigned metal salt precursors. These shortcomings generally stem from missing or oversimplified steps rather than a fundamentally incorrect sequence, suggesting that the structural integrity of the synthesis procedure is often preserved. A few cases of model hallucinations were also observed, such as repeating support synthesis mid-way, adding solids dropwise into suspensions, or instructing the user to follow the procedures from a published paper without generating the procedure itself, highlighting the current limitations of LLM-based procedure generation and underscoring the need for expert oversight.

Herein, we highlight that *HMI* is a model-centric evaluation metric applied to LLM-generated protocols and is intended to quantify how structurally and chemically coherent and actionable a generated procedure is for human review and refinement. While a greater *HMI* score generally indicates higher practical utility, we clarify that it is not meant to guarantee experimental success or certify SAC formation, especially since multiple valid synthesis routes may exist. Moreover, we note that metal-atom and support material (as queried in the synthesis model) alone do not uniquely define an SAC, since morphology-related attributes, e.g., support phase, porosity, crystallite size, surface functionalization, defect density, etc., can alter both the synthesis route and the resulting catalyst structure. Thus, our aim is not to imply a unique or correct recipe using metal-atom and support as prompts, but to test whether an LLM trained on SAC literature can generate literature-consistent protocols that follow experimental logic under user constraints, which can then be refined by an experimentalist. Learning morphology-targeted procedures would further require linking synthesis to characterization-derived structural outcomes (e.g., coordination environment), which is a promising direction but beyond the scope of the present work.

Overall, we believe generating the synthesizing procedure given the atom type and the support material is a well-defined problem for LLMs to learn and the starting point. When integrated with chemical reasoning and guided by domain experts, LLMs hold promise as foundational tools for AI-assisted synthesis planning, offering a pathway toward accelerating innovation in catalysis research.

## Conclusions

The current study presents a proof-of-concept in leveraging LLMs to generate synthesis procedures for SAC based on user-defined prompts. For this purpose, we constructed a dataset of 2964 experimental SAC-related publications spanning 2010-2024. Using this corpus, we fine-tuned the IBM Granite 3.2-8B Instruct LLM to generate SAC synthesis procedures conditioned to varying levels of user-defined inputs (e.g., metal atom, support) to fully constrained prompts, including synthesis method and the intended catalytic reaction. Our results show that instruction-tuned models can produce coherent and contextually relevant synthesis protocols, with improved performance under more detailed prompts. While LoRA-tuned models offered resource-efficient alternatives, full fine-tuning yielded greater consistency in output structure and length. Beyond metrics, we demonstrated the model’s practical value through qualitative examples and a user interface that allows researchers to query procedures tailored to their design space or resource constraints.

Importantly, we recognize and highlight that LLM-generated protocols should be viewed as assistive tools for synthesis planning in heterogeneous catalysis and not as authoritative procedures. By generating plausible synthesis protocols in response to user-defined prompts, the model lowers the barrier for experimentalists to explore alternative synthesis routes, adapt generated procedures based on their own chemical intuition, and perform model validation prior to performing the actual synthesis experiments. Aligning with this motivation, we performed a human-in-the-loop validation, wherein the domain experts evaluated the model-generated protocols for chemical feasibility and alignment with established synthesis heuristics. The approach ensures that LLM-generated synthesis procedures are not only syntactically coherent but also chemically meaningful and experimentally relevant. Such efforts will allow for scalable and human-AI collaborative synthesis planning in heterogeneous catalysis research.

## Experimental methods

### Annotation

We developed a three-step annotation methodology leveraging Qwen2.5-72B-Instruct^35^, a high-performance instruction-tuned LLM capable of zero-shot comprehension across long scientific texts. Qwen2.5-72B-Instruct is a 72-billion-parameter transformer model trained using supervised instruction-following and reinforcement learning with human feedback. It excels at multi-document comprehension, structured information extraction, and generalization across scientific domains. Its strengths include high accuracy in zero-shot and few-shot settings and the ability to handle long-context documents. However, it is computationally expensive to query, and its output may occasionally include hallucinations or require additional post-processing to resolve ambiguities in entity labeling.

The pipeline began with contextual paragraph retrieval. For each article converted from PDF to raw text through Docling, we provided the full-length article and the SI as input to Qwen2.5-72B-Instruct. The model was prompted to identify only those paragraphs containing mentions of: (i) the atomic species used in the catalyst, (ii) the support material onto which the atom is anchored, (iii) any precursor synthesis steps necessary before SAC formation, and (iv) the SAC synthesis procedure itself based on the definition described in Supplementary Table 3, and the type of catalysis being targeted (i.e., thermo-, electro-, or photocatalysis). This step was crucial because experimental procedures are often scattered across different sections of a paper. For instance, in Elsevier journals, synthesis procedures are detailed in the “Methods” section after the Introduction, whereas in most Wiley journals, synthesis information is contained in the SI, while for Springer Nature and ACS journals, the same information is detailed after the “Results” section. Thus, traditional keyword-based or rule-based approaches fail to capture such distributed context.

### Large language model development

Following the annotation and curation of the dataset, we fine-tuned the IBM Granite 3.2-8B Instruct^36^, a decoder-only transformer model pretrained for general-purpose instruction following. The 3.2-8B variant captures complex chemical language and synthesis logic while remaining computationally practical for fine-tuning on catalyst-specific data, making it a suitable choice for tasks such as generating synthesis procedures for SAC from structured prompts. We adopted two fine-tuning strategies to train the model on the annotated training set. This included (i) full fine-tuning, where all model parameters were updated during training, allowing maximal adaptation to domain-specific language and concepts, and (ii) LoRA^38^, which is a parameter-efficient approach where a small number of trainable matrices are injected into the model’s attention layers, enabling fine-tuning with significantly reduced memory and compute requirements.

We defined four distinct model variants based on the input prompts (Fig. 2a). The base model uses minimal context and is the most generic, typically requiring input on the metal species and support material. The synthesis model builds from the base counterpart by adding further information about the preferred synthesis method (Supplementary Table 3). The reaction model incorporates the most detailed prompts, including the target catalysis type, in addition to the above information. Finally, the multitask model is devised across all configurations, enabling it to respond flexibly to a range of prompt structures. All models were trained using a consistent system prompt to offer model generality (Fig. 2b and Supplementary Table 4). The multitask model was exposed to all permutations of the input fields during training, enabling it to generalize effectively to both partial and mixed prompt configurations. Training was carried out for 20 epochs with early stopping based on validation loss to prevent overfitting. Models were optimized using mixed-precision training, with a batch size of 8 and a learning rate of 2e^−5^. The best-performing checkpoint (based on the lowest validation loss) was selected for subsequent evaluation and qualitative analysis.

### Model evaluation and performance metrics

Through the study, we used a progressive model evaluation strategy ranging from semantic similarity to chemistry compliance. Specifically, we adopt three metric types in a hierarchical manner: (i) automated text-overlap metrics (initial level), (ii) automated structured procedural metrics (intermediate level), and (iii) manual human evaluation (final level).

We first use the BLEU score, Levenshtein similarity, and MLD on the test dataset. These metrics were used as text-based baselines to compare different fine-tuning variants of the model, as they provided an initial, automated quality check of whether the model outputs resemble structured procedures, and not to evaluate chemical correctness. BLEU and Levenshtein are widely used in natural language processing (NLP) studies to assess surface-level text similarity and fluency across model configurations. The BLEU score is a metric for evaluating machine-translated text, where values of 0.4-0.5 infer high-quality translations, while those below 0.25 imply poor translation and are not recommended^46,47^. Specific to this study, BLEU is used to assess n-gram overlap with the reference procedure, giving a rough sense of how similar the generated procedure is to the reference under the same prompt. Levenshtein (basic edit distance) measures how many character changes, such as insertions, deletions, or substitutions, are required to turn the generated procedure into the reference procedure. While not semantically meaningful, this represents the strictest way to assess similarity. MLD checks whether the generated procedure is about the right length. It helps identify cases where the model outputs something unnaturally short (missing steps) or too long and repetitive, serving the length as a sanity check.

The above-mentioned text-overlap metrics can remain relatively high even when key unit operations are missing, duplicated, or misplaced. To counter this challenge, we adopted a more domain-informed and structured operation-level metrics, such as: recovery of required operations (completeness), correct sequencing of matched steps (orderness), and whether steps are specified with sufficient detail to be parsed and compared (accuracy). For this purpose, we developed a custom dataset of SAC syntheses from 33 academic publications by manually annotating experimental parameters (catalyst name, synthesis method, metal, support, precursors, solvents, and procedural details), which served as the ground truth. Both the fully fine-tuned and the LoRA-tuned models were tasked with generating synthesis procedures input queries as shown in Fig. 2b. To assess correctness, we applied the ACE model, a text-to-sequence model, developed in our earlier study^21^. The ACE model converts both the generated and reference syntheses into structured representations of the experimental steps and conditions. These structured objects were then reduced to key synthesis aspects defined by the evaluation metrics defined in Section “Customized evaluation on benchmark dataset”, and the results were systematically compared against the ground truth. In this custom set, we intentionally enforce stringent matching, making the evaluation more strict compared to the test set, because synthesis protocols are sensitive to small deviations (e.g., missing washing/drying, swapping impregnation and calcination, omitting critical post-treatment conditions) that can alter the catalyst structure.

Finally, we validate the model-generated protocols through expert human evaluation^21^. Briefly, a human expert manually checks the procedures generated and grades the synthesis sequence actions on its accuracy as described in Eq. 1.

1 $$HMI=\frac{I(0)+{PC}(0.5)+C(1)}{{A}_{T}}\times 100$$

where,

*I* = incorrect actions

*PC* = partially correct actions

*C* = correct actions

*A*_T_ = total actions

The model is graded as per the *HMI* score in the following manner:

- If the model predicts the correct action terms and extracts the corresponding operational values, it gets a full score of 1.
- If the model predicts the correct action term, but extracts incorrect operational values, then it gets a score of 0.5.
- If the model predicts an action term incorrectly, it gets a score of 0.

For example, if the output of a typical SAC synthesis paragraph results in a sequence of 16 action terms with 11 correct, 2 partially correct, and 3 incorrect predictions, this results in an *HMI* of 75%. Importantly, we highlight that the *HMI* is a model-centric evaluation metric, intended to quantify how chemically coherent and actionable a generated procedure is for human review and refinement. While a higher score generally indicates higher practical utility, it is not meant to guarantee experimental success or SAC formation, especially since multiple valid synthesis routes may exist for the same metal-atom, support, and synthesis method combination.

## Supplementary information

Transparent Peer Review file

Supplementary information

## Data Availability

The curated dataset compiled in this study and the human-in-the-loop validation of the model-generated protocols are deposited at Zenodo https://doi.org/10.5281/zenodo.17989330. Further data supporting the findings of this study are available in the Supplementary Information.

## Code availability

A Python library with the preprocessing of the data and fine-tuning of the Granite model can be found on GitHub at https://github.com/rxn4chemistry/sac-finetuning. We released an open-source web application of the model at https://huggingface.co/rxn4chemistry/models/.
