# Supplementary material for: Fine-tuning large language models to generate single-atom catalyst synthesis procedures
Source: Commun Chem. 2026 Jun 19;9:313. doi: 10.1038/s42004-026-02046-y (PMC13639067; doi:10.1038/s42004-026-02046-y)
Supplement: Supplementary file 1 [file 42004_2026_2046_MOESM1_ESM.pdf]

nature portfolio

Peer Review File

Fine-tuning large language models to generate single-atom catalyst synthesis procedures

Corresponding Author: Dr Manu Suvarna

This file contains all reviewer reports in order by version, followed by all author rebuttals in order by version.

Version 0:

Reviewer comments:

Reviewer #1

(Remarks to the Author)
This paper attempts to evaluate the ability of finetuned language models to generate synthesis procedures for heterogenous catalysts. Their process, as I've understood from the paper, is as follows:

1. Curate a dataset papers from 2010-2024, and use Qwen2.5-72B-Instruct to assemble synthesis procedures from each of the papers
2. Additionally manually construct a test set of synthesis procedures from a subset of those papers, and use it to evaluate the Qwen-generated synthesis procedures
3. Train four models to generate synthesis procedures, optimized for different levels of specification on the Qwen-generated synthesis procedures
4. Evaluate these models on their similarity to procedures in the manually curated test set, in multiple ways:
1. BLEU, Levenshtein score, and mean-length difference characterization
2. machine-based structured comparisons for exact step match, order maintenance, and completeness
5. Use the manually annotated test set to discuss trends in catalyst design, and to inform later experiments about generating synthesis prompts
6. Develop a tool that serves the finetuned models to users, to allow them to explore generated potential new catalyst synthesis routes
7. Manually evaluate (fully finetuned) model predictions using the proposed HMI method to evaluate how many steps are correct or plausible.

This paper has a clear goal, and I appreciate the authors' efforts to manually validate many of the steps that go into producing this model, especially where the use of other models is concerned. The validation of the use of Qwen in the production of the training data in particular is well done, and the human evaluation of final synthesis routes is good to see, though I have some reservations with that process as I outline below. Overall, this paper has a solid core of experimental results, but has some serious issues that I'd like to see addressed. My concerns fall into a few primary buckets: concerns with the evaluation (particularly, but not exclusively, the automatic evaluation) at various points; concerns with modeling choices and potential alternatives; and insufficient specification of process that leaves unclear why certain steps were conducted, or what data looked like at certain points.

On the evaluation metrics, my first concern is with the use of BLEU, Levenshtein score, and MLD as metrics for both the Qwen annotation and assembly of synthesis procedures and for the evaluation of generated results. For the Qwen annotation (section 2.2), this choice of metrics is more understandable, given that the generated synthesis procedures are intended to be representative of the content in the paper. BLEU here does not seem like an unreasonable choice, but I would have appreciated some degree of validation that the BLEU scores were in fact indicative of successful extraction of synthesis procedures, especially given the sensitivity of these procedures to order mixups, as the authors note. MLD and Levenshtein scores are even less convincing in this context. The lengths of passages being similar can happen regardless of semantic similarity or difference, and the Levenshtein score suffers from similar issues (even aside from the fact that the authors do not specify the granularity of comparison: characters? n-grams? words?). A 0.62 similarity score does not seem very similar to me, though the procedures might be semantically equivalent. The choices of MLD and Levenshtein distance seem like low-signal metrics without validation of what they contribute to the analysis. By contrast, the use of precision, recall, and f1 for extracted content is standard, and makes sense for evaluating extracted content

These choices are much worse when evaluating generated catalyst synthesis routes from the finetuned model against the test set (section 2.3). All of these metrics rely on a closed world assumption, i.e. that any answer not present in the test set is incorrect. However, as the authors note at several points in the paper, alternate catalysts and synthesis routes are both possible and valid — an open world problem. What value, then, does the correct length of a synthesis procedure, or its Levenshtein similarity to one of potentially many correct synthesis options provide? Even in the case of BLEU score, several works [1,2, inter alia] have cast doubt on its utility, arguing that while they measure language quality reasonably, they do not measure content quality. That large language models such as those evaluated here can produce fluent text is hardly in doubt, and as such, I do not think that these metrics provide useful information as to the quality of this system, which must be measured on whether the proposed synthesis route is useful in some way, rather than being superficially statistically similar to an existing synthesis route.

The human evaluation of the generated synthesis routes (section 2.7) is therefore welcome. With this evaluation however, I'd appreciate some clarification as to how experts dealt with correctness as a property of sequences of actions, rather than individual actions, and some justification for this score being a proxy for the utility or performance of the system. For instance, if the first action in a sequence is wrong, how does that affect other actions in the sequence? How would the HMI score handle out-of-order steps? Is a synthesis procedure with several incorrect details like material properties or unit operation values still useful to the end user of this system, and does an increase in score correspond to an increase in utility?

An additional concern I have with the automated evaluation is the evaluation of the structured data from synthesis procedures (section 2.4), similarly to the issues above with the MLD and Levenshtein scores. Given the explanation in sections 2.4 and 4.4, I gather that the structured evaluation is both automatic, and primarily looking at exact match between the steps of synthesis procedures. Here, the authors themselves note that these metrics do not evaluate semantic match, and are thus likely to systematically underestimate model performance. Given this underestimate for model performance, are these metrics useful to report?

On modeling choices, I would appreciate seeing some characterization of what a non-finetuned model in the same family is capable of. I would not be surprised, given the 8B scale of the model used here, if this model did not produce valid-looking synthesis procedures; I would nonetheless appreciate the authors describing of that baseline, and what finetuning provided in addition, even if not systematically. Similarly, given that the authors fine-tune four different variants of the model, I expected to see a comparitive characterization of these models; as it stands, I am unclear which of the four models was used in the human evaluation in section 2.7.

I think it's also worth considering the effect of contamination on these scores - given that models are trained on large datasets that might include significant portions of the text used in the test set to evaluate models, could contamination have artificially inflated the scores in cases where models are reproducing knowledge from their training?

Finally, I'd appreciate some more clarity on the data curation process. I've outlined my understanding of it above, so please correct me if anything is off there, but having a table or diagram in the paper that demonstrates the paper → Qwen generated synthesis procedure → training instance curation pipeline would be greatly appreciated. As it stands, it feels like these details are scattered through the paper, and therefore hard to assemble into a coherent picture.

Overall, I think the evaluation of models for generating synthesis procedures from an automatically annotated training set with human annotation makes for a strong experimental core for this paper. With some rearranging, I think the authors could make a much more compelling case.

1] Ehud Reiter and Anja Belz. 2009. An Investigation into the Validity of Some Metrics for Automatically Evaluating Natural Language Generation Systems. Computational Linguistics, 35(4):529–558.
2] Chia-Wei Liu, Ryan Lowe, Iulian Serban, Mike Noseworthy, Laurent Charlin, and Joelle Pineau. 2016. How NOT To Evaluate Your Dialogue System: An Empirical Study of Unsupervised Evaluation Metrics for Dialogue Response Generation. In Proceedings of the 2016 Conference on Empirical Methods in Natural Language Processing, pages 2122–2132, Austin, Texas. Association for Computational Linguistics.

Reviewer #2

(Remarks to the Author)
I co-reviewed this manuscript with one of the reviewers who provided the listed reports. This is part of the Communications Chemistry initiative to facilitate training in peer review and to provide appropriate recognition for Early Career Researchers who co-review manuscripts.

Reviewer #3

(Remarks to the Author)
In this paper, the authors fine-tuned the Granite large language model among single-atom catalysis literature works. For corpus annotation, the authors used a Qwen model. For dataset curation, the authors used their fine-tuned Granite model. The authors analyzed the accuracy of the Qwen model in extracting literature information, and then the accuracy of the fine-tuned model in generating synthesis procedures. The authors also showed that suggestions on synthesizing required

catalysis could be achieved. This work showed scientific significance in advancing the field of predicting experimental procedures for SACs. However, the following concerns should be addressed as they may hurt the scientific foundation of this paper, before this paper could be published.

1. The authors reused several technics from their previous work, reference 21. However, this previous work also studied the single-atom catalysts using an LLM based on GPT-4, and presented the statistics of the synthesizing precursors. The authors' explanation of the distinction between this paper and the previous paper does not seem to be clearly explained, whereas same or similar sets of tools or conclusions could be found scattered in this paper. Therefore, to increase the scientific originality in this paper, it is recommended that the authors discuss clearly how this work is different from their previous work.

2. For data annotation, the authors annotate the data as metal atom, support material, and three other types. From experimentalist's point of view, information on the metal atom and the support material does not sufficiently determine the material, as the morphology of the material is also important. For different shape of the support material, the synthesis procedures is different. Therefore, is generating the synthesizing procedure given the atom type and the support material a well-defined problem for an LLM to learn? Could the authors comment on this question?

3. The human machine-readability index (HMI) is an interesting topic. Although the definition of HMI is provided by the authors, it is still unclear in thinking of how some subtleties are defined. For the partially correct actions, what is the meaning of "material properties" when it is annexed to "unit operation values"? Could the term "material properties", and even the word values in "unit operation values" be explained? Moreover, if the LLM outputs a sequence of 16 actions, but in reality 10 actions are needed, how will each action in 16 be scored?

4. Again about HMI, there is no objection that a better LLM on this topic should have more correct actions, but practically a successful procedure should be correct on every single action, as one wrong action or even a partially correct action in a sequence of actions where all other actions are correct may result in an invalid catalyst. Therefore, it is recommended that the authors put more careful discussions on using HMIs as evaluations.

I believe the science significance in this paper can be better revealed with the above concerns addressed.

Version 1:

Reviewer comments:

Reviewer #1

(Remarks to the Author)
Overall, I think the authors have done a good job of addressing my concerns: they have adequately delineated the scope and interpretation of their chosen metrics, and I think have made the paper stronger as a result. I do have a couple remaining nits that I would appreciate being addressed, but these are not substantive issues.

* Table 1 still does not list the base/non-finetuned model results: it still only shows fully finetuned and LoRA-based models; I still think that this comparison is important, and would appreciate the update;
* Section 2.2 interleaves descriptions of the synthesis generation model (granite-based) and the procedure extraction model (qwen-based). This section is still hard to read, and would really benefit from these two processes having their own sections. On a grammar level, at many points, e.g. the referent of "the model" is unclear.

Reviewer #2

(Remarks to the Author)
I co-reviewed this manuscript with one of the reviewers who provided the listed reports. This is part of the Communications Chemistry initiative to facilitate training in peer review and to provide appropriate recognition for Early Career Researchers who co-review manuscripts.

Reviewer #3

(Remarks to the Author)
The response from the authors very clearly addressed my questions and concerns. The science is novel and solid as far as I can see. Therefore, I recommend this paper published with no more modifications needed.

Open Access This Peer Review File is licensed under a Creative Commons Attribution 4.0 International License, which permits use, sharing, adaptation, distribution and reproduction in any medium or format, as long as you give appropriate credit to the original author(s) and the source, provide a link to the Creative Commons license, and indicate if changes were made.
In cases where reviewers are anonymous, credit should be given to 'Anonymous Referee' and the source.
The images or other third party material in this Peer Review File are included in the article's Creative Commons license, unless indicated otherwise in a credit line to the material. If material is not included in the article's Creative Commons license and your intended use is not permitted by statutory regulation or exceeds the permitted use, you will need to obtain permission directly from the copyright holder.
To view a copy of this license, visit https://creativecommons.org/licenses/by/4.0/

COMMSCHEM-26-0016 · Response to Reviewers

Comments in blue · Replies in black · Actions in **bold**

Indicated page, line, or figure numbers refer to the revised manuscript and/or supporting information with changes highlighted.

Reviewer #1

This paper attempts to evaluate the ability of finetuned language models to generate synthesis procedures for heterogenous catalysts. Their process, as I've understood from the paper, is as follows:

1. Curate a dataset papers from 2010-2024, and use Qwen2.5-72B-Instruct to assemble synthesis procedures from each of the papers
2. Additionally manually construct a test set of synthesis procedures from a subset of those papers, and use it to evaluate the Qwen-generated synthesis procedures
3. Train four models to generate synthesis procedures, optimized for different levels of specification on the Qwen-generated synthesis procedures
4. Evaluate these models on their similarity to procedures in the manually curated test set, in multiple ways:
   - BLEU, Levenshtein score, and mean-length difference characterization
   - machine-based structured comparisons for exact step match, order maintenance, and completeness
5. Use the manually annotated test set to discuss trends in catalyst design, and to inform later experiments about generating synthesis prompts
6. Develop a tool that serves the finetuned models to users, to allow them to explore generated potential new catalyst synthesis routes
7. Manually evaluate (fully finetuned) model predictions using the proposed HMI method to evaluate how many steps are correct or plausible.

This paper has a clear goal, and I appreciate the authors' efforts to manually validate many of the steps that go into producing this model, especially where the use of other models is concerned. The validation of the use of Qwen in the production of the training data in particular is well done, and the human evaluation of final synthesis routes is good to see, though I have some reservations with that process as I outline below. Overall, this paper has a solid core of experimental results, but has some serious issues that I'd like to see addressed. My concerns fall into a few primary buckets: concerns with the evaluation (particularly, but not exclusively, the automatic evaluation) at various points; concerns with modeling choices and potential alternatives; and insufficient specification of process that leaves unclear why certain steps were conducted, or what data looked like at certain points.

We sincerely thank the Reviewer for their in-depth review and summarizing our workflow, and the positive assessment of overall study, specifically mentioning the inclusion of human evaluation for model-generated synthesis routes. During the revision, we made every attempt to address the Reviewer's concerns particularly regarding the evaluation strategy (automatic and structured), and clearly rationalize our modeling choices. Below are the responses in detail.

On the evaluation metrics, my first concern is with the use of BLEU, Levenshtein score, and MLD as metrics for both the Qwen annotation and assembly of synthesis procedures and for the evaluation of generated results. For the Qwen annotation (section 2.2), this choice of metrics is more understandable, given that the generated synthesis procedures are intended to be representative of the content in the paper. BLEU here does not seem like an unreasonable choice, but I would have appreciated some degree of validation that the BLEU scores were in fact indicative of successful extraction of synthesis procedures, especially given the sensitivity of these procedures to order mixups, as the authors note. MLD and Levenshtein scores are even less convincing in this context. The lengths of passages being similar can happen regardless of semantic similarity or difference, and the Levenshtein score suffers from similar issues (even aside from the fact that the authors do not specify the granularity of comparison: characters? n-grams? words?). A 0.62 similarity score does not seem very similar to me, though the procedures might be semantically equivalent. The choices of MLD and Levenshtein distance seem like low-signal metrics without validation of what they contribute to the analysis.
By contrast, the use of precision, recall, and f1 for extracted content is standard, and makes sense for evaluating extracted content. These choices are much worse when evaluating generated catalyst synthesis routes from the finetuned model against the test set (section 2.3). All of these metrics rely on a closed world assumption, i.e. that any answer not present in the test set is incorrect. However, as the authors note at several points in the paper, alternate catalysts and synthesis routes are both possible and valid — an open world problem. What value, then, does the correct length of a synthesis procedure, or its Levenshtein similarity to one of potentially many correct synthesis options provide? Even in the case of BLEU score, several works [1,2, inter alia] have cast doubt on its utility, arguing that while they measure language quality reasonably, they do not measure content quality. That large language models such as those evaluated here can produce fluent text is hardly in doubt, and as such, I do not think that these metrics provide useful information as to the quality of this system, which must be measured on whether the proposed synthesis route is useful in some way, rather than being superficially statistically similar to an existing synthesis route.

We thank the Reviewer for the thoughtful and detailed critique. We fully agree that BLEU, Levenshtein similarity, and mean length difference (MLD) cannot establish the chemical validity or experimental soundness of generated synthesis procedures, especially in an open world domain where multiple synthesis routes may be plausible. Importantly, this was not our intended use of these metrics, for which instead the paper presents a different evaluation protocol (see point 3). Our rationale for including BLEU/Levenshtein/MLD is strictly limited to the following:

1. These metrics were used only as text based baselines to compare different fine tuning variants of the model, not to evaluate chemical correctness. BLEU and Levenshtein are widely used in

NLP to assess surface level text similarity and fluency across model configurations. Previous publications, including but not limited to *Nat. Commun.* **14**, 7964 (2023), *Nat. Commun*. **11**, 3601 (2020), have successfully used these metrics for assessing the performance of models in synthesis prediction related tasks. Their purpose in our current study was to quantify whether different fine tuning strategies lead to more coherent, procedural consistent text, rather than to judge whether the generated procedures are chemically meaningful. The latter task is left to the other evaluation protocol described in the paper (see point 3).

2. These metrics provided an initial, automated quality check of whether the model outputs resemble structured procedures at all. Given the scale of the test set (565 paragraphs), manual assessment was not feasible at this early stage. Text similarity metrics allowed us to detect degenerate outputs (e.g., overly short, repetitive, or non-procedural text), serving as an early indicator before applying more meaningful downstream evaluations.

3. Our substantive evaluation of synthesis quality does not rely on BLEU, Levenshtein, or MLD. To assess chemical plausibility and procedural logic (Sections 2.5 and 2.7), we rely instead on: (i) structured operation level metrics (step matching, ordering, completeness), which directly evaluate whether the generated procedure follows experimentally consistent actions and sequences and (ii) expert driven assessment through the HMI score, which evaluates chemical soundness, feasibility, and alignment with real laboratory practice. These are the metrics we use to draw conclusions about the model's usefulness for catalyst synthesis generation. The text level metrics appear as an initial level assessment for the model variant comparison.

The same applies on the usage of these metrics in Section 2.2, where, as the Reviewer acknowledges these metrics along with precision, recall, and f1 score is more meaningful.

To address the Reviewer's concern that these metrics may be low-signal without clear definitions, we have clarified in the manuscript that:

- BLEU is used to assess n-gram overlap with the reference procedure giving a rough sense of how similar the generated procedure is to the reference under the same prompt.
- Levenshtein (basic edit distance) measures how many character changes such as insertions, deletions, or substitutions are required to turn the generated procedure into the reference procedure. While not semantically meaningful this represent the strictest way to assess similarity.
- MLD checks whether the generated procedure is about the right length. It helps identify cases where the model outputs something unnaturally short (missing steps) or too long and repetitive, serving the length as a sanity check.

We have strengthened these discussions in the revised manuscript in page 8, lines 12-18, page 20, lines 11-28, and page 21, lines 1-26, to make our rationale explicit and avoid any over-claim from BLEU/Levenshtein/MLD scores.

The human evaluation of the generated synthesis routes (section 2.7) is therefore welcome. With this evaluation however, I'd appreciate some clarification as to how experts dealt with correctness as a property of sequences of actions, rather than individual actions, and some justification for this score being a proxy for the utility or performance of the system. For instance, if the first action in a sequence is wrong, how does that affect other actions in the sequence? How would the HMI score handle out-of-order steps? Is a synthesis procedure with several incorrect details like material properties or unit operation values still useful to the end user of this system, and does an increase in score correspond to an increase in utility?

We thank the Reviewer for this important question on Human Machine-Readability Index (HMI) scoring (also aligned with Reviewer #3, Comments 3 and 4). The HMI is a model-centric evaluation metric applied to LLM-generated protocols, and is intended to quantify how structurally and chemically coherent and actionable a generated procedure is for human review and refinement. While higher HMI generally indicates higher practical utility, **we clarify that HMI is not meant to guarantee experimental success or certify SAC formation, in page 22, lines 18-23 in the revised manuscript**.

Regarding the scoring, HMI assigns scores at the step level, and incorrect steps are penalized even if later steps appear plausible. For example, if an LLM generates 6 steps and the first step is incorrect while the remaining 5 are correct, the score would be 5/6 under the rubric (0 for step#1, and 1 each for steps#2-6). We agree that an experimentally incorrect first step can compromise the entire synthesis, (such early-step errors were rare in our human validation (<5%)), and we emphasize that the model outputs should not be followed blindly and expert oversight is required as incorrect steps can be corrected by an experimentalist on need basis. Similarly missing/incorrect unit-operation parameters (e.g., temperature, solvent concentration, drying time etc) are also penalized, since these affect whether a protocol is actionable.

To this aim, we present a case-study outlining the various scenarios listed by the Reviewer and how the HMI scoring would take effect and rationalize the scoring approach. **These are discussed in Supplementary Note 3 in the revised supplementary information**.

An additional concern I have with the automated evaluation is the evaluation of the structured data from synthesis procedures (section 2.4), similarly to the issues above with the MLD and Levenshtein scores. Given the explanation in sections 2.4 and 4.4, I gather that the structured evaluation is both automatic, and primarily looking at exact match between the steps of synthesis procedures. Here, the authors themselves note that these metrics do not evaluate semantic match, and are thus likely to systematically underestimate model performance. Given this underestimate for model performance, are these metrics useful to report?

We agree with the Reviewer that the structured metrics in Section 2.4 (accuracy, orderness, completeness) may not capture full semantic equivalence and can therefore underestimate performance in an open-world setting where multiple valid routes, paraphrases, or step granularities may exist. Nevertheless, we retain these metrics because they quantify domain-relevant validation. Every paragraph in this custom set was manually annotated for synthesis

actions and operating parameters. In this custom set, we intentionally enforce stringent matching, making the evaluation more strict compared to the test set, because synthesis protocols are sensitive to small deviations (e.g., missing washing/drying, swapping impregnation and calcination, omitting critical post-treatment conditions) can materially alter the catalyst structure. Under such strict criteria, low-to-moderate scores are expected and are best interpreted as a lower bound on exact procedural reproduction, not as a final estimate of open-world correctness. **In the revised manuscript, we discuss these aspects in page 9, lines 10-14 and page 10, lines 19-27.**

On the other hand, text-overlap scores (BLEU, Levenshtein, MLD) can remain relatively high even when key unit operations are missing, duplicated, or misplaced. Thus, structured metrics explicitly allow procedure-defining elements: recovery of required operations (completeness), correct sequencing of matched steps (orderness), and whether steps are specified with sufficient detail to be parsed and compared (accuracy).

We agree with the Reviewer that ultimate usefulness must be assessed through expert judgment of experimental logic. Accordingly, we use progressive evaluation strategy: (i) text-overlap metrics (initial level), (ii) structured procedural metrics (intermediate level), and (iii) HMI/human evaluation (experimental coherence). We recognize this overall rationale on evaluation metrics selection was not captured in the initial submission. **In the revised manuscript we make this hierarchy explicit in page 20, lines 11-14.**

On modeling choices, I would appreciate seeing some characterization of what a non-finetuned model in the same family is capable of. I would not be surprised, given the 8B scale of the model used here, if this model did not produce valid-looking synthesis procedures; I would nonetheless appreciate the authors describing of that baseline, and what finetuning provided in addition, even if not systematically. Similarly, given that the authors fine-tune four different variants of the model, I expected to see a comparative characterization of these models; as it stands, I am unclear which of the four models was used in the human evaluation in section 2.7.

We thank the Reviewer for the suggestion. In the revised manuscript, **we now report the performance of the corresponding non-finetuned model in Table 1.** This comparison clearly highlights that both fine-tuning strategies (full fine-tuning and LoRA) lead to substantial improvements over the non fine-tuned model, and Table 1 also includes the results for all four model variants considered, enabling a direct comparison across the different prompt formats used for the tuning experiments.
For the human validation, we utilize the fully-fine tuned using the synthesis-model, as this model directly catered to the requirement. In the revised version, **we explicitly specify this detail in page 14, lines 6-8.**

I think it's also worth considering the effect of contamination on these scores - given that models are trained on large datasets that might include significant portions of the text used in the test set to evaluate models, could contamination have artificially inflated the scores in cases where models are reproducing knowledge from their training?

Regarding potential contamination, we acknowledge that we cannot categorically rule out the possibility that some portions of the evaluation papers appeared in the large-scale pre-training data used for the base model. However, the consistent performance gains observed after fine-tuning indicate that any such contamination, if present, plays only a minor role in the reported results. Moreover, the evaluation set comprises a small number of papers relative to the scale of the pre-training corpora, making any contamination effect negligible in practice.

Finally, I'd appreciate some more clarity on the data curation process. I've outlined my understanding of it above, so please correct me if anything is off there, but having a table or diagram in the paper that demonstrates the paper → Qwen generated synthesis procedure → training instance curation pipeline would be greatly appreciated. As it stands, it feels like these details are scattered through the paper, and therefore hard to assemble into a coherent picture.

We thank the Reviewer for the suggestion and their understanding on the data curation and modelling, as outlined at the start is correct. Following the suggestion, and to provide clarity to the readers, **we present a graphical flow chart of the data curation and modelling logic in Figure 1a.**

Overall, I think the evaluation of models for generating synthesis procedures from an automatically annotated training set with human annotation makes for a strong experimental core for this paper. With some rearranging, I think the authors could make a much more compelling case.

1] Ehud Reiter and Anja Belz. 2009. An Investigation into the Validity of Some Metrics for Automatically Evaluating Natural Language Generation Systems. Computational Linguistics, 35(4):529–558.
2] Chia-Wei Liu, Ryan Lowe, Iulian Serban, Mike Noseworthy, Laurent Charlin, and Joelle Pineau. 2016. How NOT To Evaluate Your Dialogue System: An Empirical Study of Unsupervised Evaluation Metrics for Dialogue Response Generation. In Proceedings of the 2016 Conference on Empirical Methods in Natural Language Processing, pages 2122–2132, Austin, Texas. Association for Computational Linguistics.

We thank the Reviewer for their constructive suggestions. We have revised the manuscript accordingly, and we believe the changes prompted by their comments helped improve the clarity, rigor, and presentation of the work.

Reviewer #2

I co-reviewed this manuscript with one of the Reviewers who provided the listed reports. This is part of the Communications Chemistry initiative to facilitate training in peer review and to provide appropriate recognition for Early Career Researchers who co-review manuscripts.

We appreciate and thank the Reviewer, for their time to co-review the manuscript along with the other Reviewer.

Reviewer #3

In this paper, the authors fine-tuned the Granite large language model among single-atom catalysis literature works. For corpus annotation, the authors used a Qwen model. For dataset curation, the authors used their fine-tuned Granite model. The authors analyzed the accuracy of the Qwen model in extracting literature information, and then the accuracy of the fine-tuned model in generating synthesis procedures. The authors also showed that suggestions on synthesizing required catalysis could be achieved. This work showed scientific significance in advancing the field of predicting experimental procedures for SACs. However, the following concerns should be addressed as they may hurt the scientific foundation of this paper, before this paper could be published.

We sincerely thank Reviewer #3 for their constructive feedback and positive assessment of the manuscript. We agree the points raised are important for strengthening the scientific foundation and clarity of the manuscript. Below we present point-by-point response and describe the corresponding revisions.

1. The authors reused several technics from their previous work, (reference 21). However, this previous work also studied the single-atom catalysts using an LLM based on GPT-4, and presented the statistics of the synthesizing precursors. The authors' explanation of the distinction between this paper and the previous paper does not seem to be clearly explained, whereas same or similar sets of tools or conclusions could be found scattered in this paper. Therefore, to increase the scientific originality in this paper, it is recommended that the authors discuss clearly how this work is different from their previous work.

We thank the Reviewer for the opportunity to clarify the distinction between the current manuscript and our prior work *Nat. Commun.* **14**, 7964 (2023).
Firstly, the core motivation and task is different in both the studies. Our previous work is non-generative in nature, i.e. the user provides an existing full synthesis procedure in free text, and the transformer model performs a text-to-sequence breakdown into structured synthesis actions. In contrast, the present work is a clear step forward in the topic of AI-assisted synthesis planning, wherein we fine-tune an LLM on SAC recipes curated from the literature to generate synthesis procedures de novo, based on user-defined prompts (e.g., metal-support combination, synthesis method, and target reaction). This shift from procedure interpretation to procedure generation is a substantially more challenging task, and the main conceptual advance
Furthermore, the scientific emphasis also differs in both the studies. In *Nat. Commun.* **14**, 7964 (2023) we primarily highlighted the need for data standardization to enable reliable text-to-sequence conversion. Here, we focus on trustworthiness of LLM-generated recipes, using multiple automated and human-in-the-loop evaluation metrics to test whether generated protocols are coherent, context-aware, and consistent with experimental logic rather than hallucinated. **We agree these distinctions should be explicitly stated, and include the same in the Introduction section, in page 3, lines 9-20.**

2. For data annotation, the authors annotate the data as metal atom, support material, and three other types. From experimentalist's point of view, information on the metal atom and the support material does not sufficiently determine the material, as the morphology of the material is also important. For different shape of the support material, the synthesis procedures is different. Therefore, is generating the synthesizing procedure given the atom type and the support material a well-defined problem for an LLM to learn? Could the authors comment on this question?

We agree with the Reviewer's perspective that metal identity and support material alone does not determine an SAC morphology (e.g., support phase, porosity, crystallite size, surface functionalization, defect density) and its function. We also acknowledge, synthesis procedures are largely chosen keeping the metal atoms and support in mind, which in turn dictates the resulting catalyst structure.

Our intent in this work is not to claim the synthesis procedure is only determined by metal atom and support alone, but rather to study whether an LLM trained on SAC procedures can generate a chemically coherent protocols consistent with experimental logic reported in literature. We believe this is the first step and an interesting problem for LLMs to learn as there are many synthesis procedures reported in literature. We also considered more advanced ideas, such as, could LLMs generate SAC structures with defined morphologies (specific co-ordination sites and functional group). This will require LLMs not only to learn synthesis, but also the corresponding characterization section of the paper, mapping synthesis to detailed structure and beyond the scope of current work. To address the well-posed concern, **we rationalize our motivation and further discuss the possibilities and limitations of LLMs to generate SAC synthesis procedures in page 16, lines 4-13.**

3. The human machine-readability index (HMI) is an interesting topic. Although the definition of HMI is provided by the authors, it is still unclear in thinking of how some subtleties are defined. For the partially correct actions, what is the meaning of "material properties" when it is annexed to "unit operation values"? Could the term "material properties", and even the word values in "unit operation values" be explained? Moreover, if the LLM outputs a sequence of 16 actions, but in reality 10 actions are needed, how will each action in 16 be scored?

We thank the Reviewer for highlighting these ambiguities. In our framework, each procedure step is defined by an action term (e.g., dissolve, impregnate, dry, calcine) and its corresponding parametric value (called as unit operation values), such as temperature, time, atmosphere, solvent concentration, precursor amount, cooling or heating temperature, stirring speed, pH, etc. To avoid confusion, we have revised the terminology and discarded the term "material properties" and hereon use the term "operational values" only. This should make clear that the "partially correct" category refers to cases where the action term is correct but some operation parameters are wrong or missing or incomplete.

In the current Human Machine-Readability Index (HMI) implementation, the generated procedure is the scoring basis. For example, if the LLM outputs 16 synthesis steps - all of them are evaluated, even if only 10 steps are required for the same or alternate synthesis. Each of the 16 step is

evaluated by the domain expert, according to the correctness or incorrectness based on chemistry plausibility, which makes HMI deterministic. At the same time, we agree that in real world cases, multiple valid routes can exist for the same target material, often resulting in procedures with different numbers of steps (also discussed in our response to Reviewer #1). Accordingly, we interpret HMI as a model-centric evaluation metric for evaluating LLM-generated protocols and not as proxies for experimental success. **In the revision manuscript we clarify the terminology aspect in page 22, lines 11-14. We also showcase examples on the scoring of the HMI under various scenarios in Supplementary Note 3 in the revised supplementary information**.

4. Again about HMI, there is no objection that a better LLM on this topic should have more correct actions, but practically a successful procedure should be correct on every single action, as one wrong action or even a partially correct action in a sequence of actions where all other actions are correct may result in an invalid catalyst. Therefore, it is recommended that the authors put more careful discussions on using HMIs as evaluations.

We thank the Reviewer for this important question on HMI scoring which builds from the above comment (and also aligned with Reviewer #1, Comment #2). The HMI is a model-centric evaluation metric applied to LLM-generated protocols, and is intended to quantify how structurally and chemically coherent and actionable a generated procedure is for human review and refinement. While higher HMI generally indicates higher practical utility, we clarify that HMI is not meant to guarantee experimental success or certify SAC formation, especially since multiple valid synthesis routes may exist.

Regarding the scoring, HMI assigns scores at the step level, and incorrect steps are penalized even if later steps appear plausible. For example, if an LLM generates 6 steps and the first step is incorrect while the remaining 5 are correct, the score would be 5/6 under the rubric (0 for step#1, and 1 each for steps#2-6). We agree that an experimentally incorrect first step can compromise the entire synthesis, (such early-step errors were rare in our human validation (<5%)), and we emphasize that the model outputs should not be followed blindly and expert oversight is required as incorrect steps can be corrected by an experimentalist on need basis. Similarly missing/incorrect unit-operation parameters (e.g., temperature, solvent concentration, drying time etc) are also penalized, since these affect whether a protocol is actionable.

To this aim, we present a case-study outlining the various scenarios listed by the Reviewer and how the HMI scoring would take effect and rationalize the scoring approach. **These are discussed in page 22, lines 18-23 and in Supplementary Note 3 in the revised supplementary information**.

COMMSCHEM-26-0016A · Response to Reviewers

Comments in blue · Replies in black · Actions in **bold**

Indicated page, line, or figure numbers refer to the revised manuscript and/or supporting information with changes highlighted.

Reviewer #1

Overall, I think the authors have done a good job of addressing my concerns: they have adequately delineated the scope and interpretation of their chosen metrics, and I think have made the paper stronger as a result. I do have a couple remaining nits that I would appreciate being addressed, but these are not substantive issues.

We sincerely thank the Reviewer for their in-depth peer-review and comments which helped us further strengthen the paper. We also thank them for supporting the acceptance of the paper. In the final revised version, we made necessary changes as per the suggestions.

Table 1 still does not list the base/non-finetuned model results: it still only shows fully finetuned and LoRA-based models; I still think that this comparison is important, and would appreciate the updates.

Table 1 includes the results of the non-fine-tuned model in parentheses. A footnote has been listed to guide the readers on the same.

Section 2.2 interleaves descriptions of the synthesis generation model (granite-based) and the procedure extraction model (qwen-based). This section is still hard to read, and would really benefit from these two processes having their own sections. On a grammar level, at many points, e.g. the referent of “the model” is unclear.

We thank the suggestion and have further divided Section 2.2 into two subsections, one each for the Granite model and the other for the Qwen model. This should help clarify the message to the readers.

Reviewer #2

I co-reviewed this manuscript with one of the Reviewers who provided the listed reports. This is part of the Communications Chemistry initiative to facilitate training in peer review and to provide appropriate recognition for Early Career Researchers who co-review manuscripts.

We appreciate and thank the Reviewer, for their time to co-review the manuscript along with the other Reviewer.

Reviewer #3

The response from the authors very clearly addressed my questions and concerns. The science is novel and solid as far as I can see. Therefore, I recommend this paper published with no more modifications needed.

We sincerely thank Reviewer #3 for their constructive feedback and positive assessment of the manuscript and supporting its publication.
