# Supplementary material for: Fine-tuning large language models to generate single-atom catalyst synthesis procedures
Source: Commun Chem. 2026 Jun 19;9:313. doi: 10.1038/s42004-026-02046-y (PMC13639067; doi:10.1038/s42004-026-02046-y)
Supplement: Supplementary file 2 [file 42004_2026_2046_MOESM2_ESM.pdf]

Fine-tuning large language models to generate single-atom catalyst synthesis procedures

Manu Suvarna[1,4,*], Matteo Manica[2], Fillipo Ficarra[2], Andres M. Bran[3], Andrea Ruiz-Ferrando[1,4], Magdalena Lederbauer[3], Philippe Schwaller[3,4], Javier Pérez-Ramírez[1,4], Teodoro Laino[2,4]

[1]Institute for Chemical and Bioengineering, Department of Chemistry and Applied Biosciences, ETH Zurich, Vladimir-Prelog-Weg 1, 8093 Zurich, Switzerland.

[2]IBM Research Europe, Säumerstrasse 4, 8803 Rüschlikon, Switzerland.

[3]Laboratory of Artificial Chemical Intelligence, Institute of Chemical Sciences and Engineering, EPFL, 1015 Lausanne, Switzerland.

[4]NCCR Catalysis, 8093 Zurich, Switzerland.

*Corresponding author. E-mail: msuvarna@chem.ethz.ch, s.suvarnamanu@gmail.com

Supplementary Information

Supplementary Note 1. Extraction accuracy of the Qwen2.5-72B-Instruct model

We used three metrics to quantify the extraction accuracy of the Qwen2.5-72B-Instruct model.[1,2] The Levenshtein similarity, a measure of how similar two pieces of text are by calculating the number of edits (insertions, deletions, or substitutions) required to turn one into the other. A similarity score of 0.62 (on a scale from 0 to 1) indicates that, on average, more than half of the text matched between the generated and reference procedures. Specifically in our study, this implies the model reproduced the key synthesis steps and terminology reasonably well, although differences in phrasing or level of detail can vary the score. We also examined the BiLingual Evaluation Understudy (BLEU) score, where a score of 0.51 shows moderate to significant overlap in the chemistry vocabulary and procedural language used by the model, even if the exact word order varies. For example, 'heat at 300°C for 2 h' and 'maintain 300°C for 2 h under heating' may differ syntactically but share the same information from a chemistry point of view. Lastly, we calculated the mean length difference (MLD) score, where an average value of 0.023 indicates that the model-generated procedures were nearly the same length as the references. This suggests the model is neither skipping critical steps nor over-generating irrelevant content important for preserving the completeness of synthetic workflows. However, we caution that MLD does not account for semantic accuracy as two procedures of equal length may still differ significantly in meaning or synthesis validity. This limitation is partially mitigated by the complementary insights provided by the Levenshtein and BLEU scores.

Supplementary Note 2. Evaluation of categorical information extraction accuracy

We evaluated the LLM's capability to accurately extract structured categorical attributes, namely the catalysis type and synthesis route for each SAC protocol. Since this analysis was classification in nature, model performance was quantified using metrics inlcuding accuracy, recall, and precision. Recall is the fraction of relevant instances correctly captured and precision is the fraction of correct predictions among all outputs. These metrics are defined in **Equations 1-3**,

$$\text{Accuracy} = \frac{\text{TP} + \text{TN}}{\text{TP} + \text{FP} + \text{TN} + \text{FN}} \tag{1}$$

$$\text{Precision} = \frac{\text{TP}}{\text{TP} + \text{FP}} \tag{2}$$

$$\text{Recall} = \frac{\text{TP}}{\text{TP} + \text{FN}} \tag{3}$$

where TP, TN, FP, and FN are the true positive, true negative, false positive, and false negatives respectively.

Supplementary Note 3. Overview of the *HMI* scoring by the domain expert

In this section, we illustrate several representative scenarios to show how a domain expert manually evaluated synthesis procedures generated by the synthesis variant of the fully fine-tuned model. For each scenario, the same prompt was used, and the model generated a seven-step synthesis procedure. The step-wise scores and the corresponding expert rationale (in *italics*) are provided for each case.

Prompt: You are a helpful assistant. Given the following information, provide the user with the procedure to synthesize single-atom catalyst/
Metal atom: Iron
Support material: Ceria
Synthesis method: Hybrid solution-high temperature.

Scenario 1 – Fully correct protocol

- Step 1: 1g of cerium (II) nitrate hexahydrate was dissolved in 30 mL of deionized water and then transferred to a Teflon-lined stainless steel autoclave and placed in an oven at 180 °C for 24 h.
  Score: 1 point
  Rationale: *Preparation of the support material with correct precursor, solution making steps, with correct unit operations and their respective operational value*
- Step 2: The precipitate was collected by centrifugation, washed with deionized water three times and then dried at 60°C overnight.
  Score: 1 point
  Rationale: *Correct unit operation with operational value*
- Step 3: Finally, the powder was calcined at 500 °C for 2 h under air.
  Score: 1 point
  Rationale: *Correct thermal treatment with operational value*
- Step 4: 10 mg of iron (III) acetylacetonate was dissolved into 30 mL ethanol solution and sonicated for 30 min.
  Score: 1 point
  Rationale: *Preparation of the metal precursor solution with correct details*
- Step 5: Then, 1.0 g of CeO2 support was added to the solution, followed by stirring at 300 rpm for 12 h at room temperature
  Score: 1 point
  Rationale: *Metal-support impregnation with correct details*
- Step 6: After that, the precipitate was separated from the solution using centrifugation, washed with deionized water several times, and dried at 60 °C for 12 h.
  Score: 1 point
  Rationale: *Correct unit operation with operational value*
- Step 7: Finally, the sample was calcined at 300 °C for 1 h under air.
  Score: 1 point
  Rationale: *Correct thermal treatment step with operational value*

The total score of this procedure is 7/7 achieving a perfect score as all the synthesis steps and the operational values are correct.

Scenario 2 – Partially correct protocol

- Step 1: 1g of cerium (II) nitrate hexahydrate was dissolved in 30 mL of deionized water and then transferred to a Teflon-lined stainless steel autoclave and placed in an oven at 180 °C for 24 h.
  Score: 1 point
  Rationale: *Preparation of the support material with correct precursor, solution making steps, with correct unit operations and their respective operational value*
- Step 2: The precipitate was collected by centrifugation, washed with deionized water three times and then dried at 60°C overnight.
  Score: 1 point
  Rationale: *Correct unit operation with operational value*
- Step 3: Finally, the powder was calcined
  Score: 0.5 point
  Rationale: *Correct thermal treatment with but the operational value is missing. Hence this steps gets only 0.5 points for listing the action correctly.*
- Step 4: 10 mg of iron (III) acetylacetonate was dissolved into 30 mL ethanol solution and sonicated for 30 min.
  Score: 1 point
  Rationale: *Preparation of the metal precursor solution with correct details*
- Step 5: Then, 1.0 g of CeO2 support was added to the solution, followed by stirring at 300 rpm for 12 h at room temperature
  Score: 1 point
  Rationale: *Metal-support impregnation with correct details*
- Step 6: After that, the precipitate was separated from the solution using centrifugation, washed with deionized water several times, and dried.
  Score: 0.5 point
  Rationale: *Correct series of unit operations but the operational values are missing. Hence this steps gets only 0.5 points for listing the action correctly.*
- Step 7: Finally, the sample was calcined at 300 °C for 1 h under air.
  Score: 1 point
  Rationale: *Correct thermal treatment step with operational value*

The total score of this procedure is 6/7. It contains five fully correct steps achieving 1 point each and two partially correct steps achieving 0.5 point each.

Scenario 3 – Partially correct protocols with incorrect chemistry

- Step 1: 1g of cerium (II) nitrate hexahydrate was dissolved in 30 mL of deionized water and then transferred to a Teflon-lined stainless steel autoclave and placed in an oven at 180 °C for 24 h.
  Score: 1 point
  Rationale: *Preparation of the support material with correct precursor, solution making steps, with correct unit operations and their respective operational value*
- Step 2: The precipitate was collected by centrifugation, washed with deionized water three times and then dried at 60°C overnight.
  Score: 1 point
  Rationale: *Correct unit operation with operational value*
- Step 3: Finally, the powder was calcined.
  Score: 0.5 point
  Rationale: *Correct thermal treatment with but the operational value is missing. Hence this steps gets only 0.5 points for listing the action correctly.*
- Step 4: 10 mg of platinum acetate was dissolved into 30 mL ethanol solution and sonicated for 30 min.
  Score: 0 point
  Rationale: *Incorrect metal precursor used*
- Step 5: Then, 1.0 g of CeO2 support was added to the solution, followed by stirring at 300 rpm for 12 h at room temperature
  Score: 1 point
  Rationale: *Metal-support impregnation step with correct details*
- Step 6: After that, the precipitate was separated from the solution using centrifugation, washed with deionized water several times, and dried.
  Score: 0.5 point
  Rationale: *Correct series of unit operations but the operational values are missing. Hence this steps gets only 0.5 points for listing the action correctly.*
- Step 7: Finally, the sample was calcined at 300°C for 1 h under air.
  Score: 1 point
  Rationale: *Correct thermal treatment step with operational value*

The total score of this procedure is 5/7. The total score of this procedure is 6/7. It contains four fully correct steps achieving 1 point each, two partially correct steps achieving 0.5 point each, and one incorrect step. While it is important to acknowledge that the use of an incorrect metal precursor e.g., platinum acetate instead of an iron precursor, would fundamentally change the target SAC and render the synthesis unsuccessful, *HMI* is intended as a model-centric diagnostic metric. It evaluates how chemically coherent and actionable a generated procedure is for human review and refinement. Accordingly, if one step is incorrect, whether at the beginning or in the middle of the sequence, we do not disqualify the remaining steps, since a

domain expert may still identify the error and take corrective action while assessing the rest of the protocol.

Supplementary Table 1. Accuracy metrics for catalysis type on extraction evaluation.

**Catalysis type**	**Recall**	**Precision**
Thermo	0.54	0.81
Electro	0.92	0.83
Photo	1.00	0.87

Supplementary Table 2. Accuracy metrics for synthesis type on extraction evaluation.

**Synthesis method**	**Recall**	**Precision**
Hybrid high-temperature solution phase	0.88	0.63
Photochemical	0.83	0.62
Hybrid electrochemical high-temperature	0.33	1.00
Electrochemical	1.00	0.50
Vapour/gas-phase	0.43	0.75
Solution phase	0.17	0.67
Mechanochemical	0.00	0.00
High temperature	0.00	0.00

Supplementary Table 3. Classification of single-atom catalysts synthetic methods.[3-5]

Synthetic methods	Approaches covered	Description
High-temperature synthesis	• Pyrolysis<br>• Thermal decomposition<br>• Carbonization<br>• Polymerization	Synthesis of SAC by thermal treatment (*T*>573 K).
Gas and vapor phase synthesis	• Chemical vapor deposition<br>• Physical vapor deposition<br>• Molecular beam epitaxy<br>• Pulsed laser deposition	Synthesis of SAC in the gas phase or by deposition of a vaporized precursor.
Solution-phase synthesis	• (Wet/dry) impregnation<br>• Wet deposition<br>• Ion exchange<br>• Precipitation<br>• Hydrothermal<br>• Colloidal synthesis	Synthesis of SAC in solution phase
Electrochemical synthesis	• Electrodeposition<br>• Galvanic displacement	Synthesis of SAC through electrochemical reactions.
Photochemical synthesis	• Photoreduction<br>• Photodeposition<br>• Photocatalytic	Synthesis of SAC using light as a source of energy.
Mechanochemical synthesis	• Grinding<br>• Milling<br>• Shearing	Synthesis of SAC using mechanical energy.
Templated synthesis	• Molecular templates<br>• Porous material templates	Uses templates to guide the atomic dispersion of the metal or tailor the properties, such as porosity, of the support.
Hybrid synthesis	• For example, high-temperature and solution phase	Combines more than one synthetic approach.
Post-synthetic treatments	• Oxidation<br>• Reduction<br>• Heating/Drying<br>• Annealing<br>• Acid leaching<br>• Support modification<br>• Addition of promoter	Refer to processes applied after synthesis to tune the SAC properties or remove unwanted species (metal clusters or byproducts). While they are separate steps from synthesis, they are often interdependent.

Supplementary Table 4. Prompts used to train the models with full fine-tuning and LoRA. The model denoted as multitask was trained on all the different prompts together.

**Model**	**Prompt**
Base	• Atom<br>• Support Material
Synthesis	• Atom<br>• Support Material<br>• Synthesis Method
Reaction	• Atom<br>• Support Material<br>• Synthesis Method<br>• Catalysis Type
Multitask	• Atom<br>• Support Material<br><br>• Atom<br>• Support Material<br>• Synthesis Method<br><br>• Atom<br>• Support Material<br>• Synthesis Method<br>• Catalysis Type<br><br>• Atom<br>• Synthesis Method<br><br>• Atom<br>• Catalysis Type<br><br>• Atom<br>• Support Material<br>• Catalysis Type<br><br>• Atom<br>• Synthesis Method<br>• Catalysis Type

References

1. Vaucher, A. C. *et al.* Automated extraction of chemical synthesis actions from experimental procedures. *Nat. Commun.* **11**, 3601 (2020).
2. Suvarna, M., Vaucher, A. C., Mitchell, S., Laino, T. & Pérez-Ramírez, J. Language models and protocol standardization guidelines for accelerating synthesis planning in heterogeneous catalysis. *Nat. Commun.* **14**, 7964 (2023).
3. Ji, S. *et al.* Chemical synthesis of single atomic site catalysts. *Chem. Rev.* **120**, 11900–11955 (2020).
4. Guo, W., Wang, Z., Wang, X. & Wu, Y. General design concept for single-atom catalysts toward heterogeneous catalysis. *Adv. Mater.* **33**, 2004287 (2021).
5. Chen, Y. *et al.* Single-atom catalysts: synthetic strategies and electrochemical applications. *Joule* **2**, 1242–1264 (2018).
